# The complex impact of cancer-related missense mutations on the stability and on the biophysical and biochemical properties of MAPK1 and MAPK3 somatic variants

**DOI:** 10.1186/s40246-023-00544-x

**Published:** 2023-10-27

**Authors:** Maria Petrosino, Leonore Novak, Alessandra Pasquo, Paola Turina, Emidio Capriotti, Velia Minicozzi, Valerio Consalvi, Roberta Chiaraluce

**Affiliations:** 1 Chair of Pharmacology, Section of Medicine, University of Fribourg, Fribourg, Switzerland; 2 Dipartimento di Scienze Biochimiche “A. Rossi Fanelli”, Sapienza University of Rome, Rome, Italy; 3 ENEA CR Frascati, Diagnostics and Metrology Laboratory FSN-TECFIS-DIM, Frascati, Italy; 4 Department of Pharmacy and Biotechnology, University of Bologna, Bologna, Italy; 5 Department of Physics, University of Rome Tor Vergata, Via della Ricerca Scientifica 1, 00133 Rome, Italy

## Abstract

Mitogen-activated protein kinases 1 and 3 (MAPK1 and MAPK3), also called extracellular regulated kinases (ERK2 and ERK1), are serine/threonine kinase activated downstream by the Ras/Raf/MEK/ERK signal transduction cascade that regulates a variety of cellular processes. A dysregulation of MAPK cascade is frequently associated to missense mutations on its protein components and may be related to many pathologies, including cancer. In this study we selected from COSMIC database a set of MAPK1 and MAPK3 somatic variants found in cancer tissues carrying missense mutations distributed all over the MAPK1 and MAPK3 sequences. The proteins were expressed as pure recombinant proteins, and their biochemical and biophysical properties have been studied in comparison with the wild type. The missense mutations lead to changes in the tertiary arrangements of all the variants. The thermodynamic stability of the wild type and variants has been investigated in the non-phosphorylated and in the phosphorylated form. Significant differences in the thermal stabilities of most of the variants have been observed, as well as changes in the catalytic efficiencies. The energetics of the catalytic reaction is affected for all the variants for both the MAPK proteins. The stability changes and the variation in the enzyme catalysis observed for most of MAPK1/3 variants suggest that a local change in a residue, distant from the catalytic site, may have long-distance effects that reflect globally on enzyme stability and functions.

**Supplementary Information:**

The online version contains supplementary material available at 10.1186/s40246-023-00544-x.

## Introduction

Over five hundreds different protein kinases are forming the human kinome, one of the largest gene families in eukaryotes, and are involved in a wide range of cell signaling processes and, therefore, also in several human diseases [23]. Mitogen-activated protein kinases 1 and 3 (MAPK1 and MAPK3), also called extracellular regulated kinases (ERK2 and ERK1), in the kinase tree [20] belong to the evolutionary conserved group of CMGC: cyclin-dependent kinases (CDKs), mitogen-activated protein kinases (MAPKs), glycogen synthase kinases (GSKs), and CDK-like kinases (CLKs) [5]. The collectively termed CMGC group is formed by 62 members (http://uniprot.org), assigned to nine families. The CDKs and MAPKs are the two largest and best studied in the CMGC groups, since CDKs control the activity of human tumor suppressors and MAPKs regulate a variety of cellular processes and participate extensively in the control of cell-fate decisions across all the eukaryotic phyla [46].

The MAPK signaling cascade, Ras/Raf/MEK/ERK or ERK1/2 cascade, is a central pathway where the extracellular signal is propagated by sequential phosphorylation and activations of the sequential kinases. The signal of this protein cascade terminates with the phosphorylation of target proteins that are localized in cytoplasm, mitochondria, Golgi, ER, and in the nucleus where the cascade may induce and/or regulate de-novo gene expression [30]. The phosphorylation signal regulates and controls many fundamental cellular processes, including growth, proliferation, differentiation, stress response and motility, and finally survival or apoptosis [17]. Notably, a dysregulation of MAPK cascade may be involved in many pathological situations [18], mainly cancers [34] but also neurodegenerative [15] or autoimmune disease [19], and diabetes [25]. The MAPK cascade dysregulation is frequently associated to point mutations, due to single nucleotide substitutions, on the proteins component of the signaling cascade [16]. Because of their biological importance, MAPK cascade represents an important target of biomedical research and of drug discovery research [33, 48].

MAPK1 and MAPK3 are serine/threonine kinase activated downstream by the Ras/Raf/MEK/ERK signal transduction cascade, where they represent the last step in the signaling cascade. The effector kinases MAPK1 and MAPK3 are isoforms encoded by distinct genes and in humans are located on two different chromosomes, 22q11 and 16q11, respectively. Their relative abundance is considerably different in various tissues and cell types though they are always co-expressed [37].

**Fig. 1:**
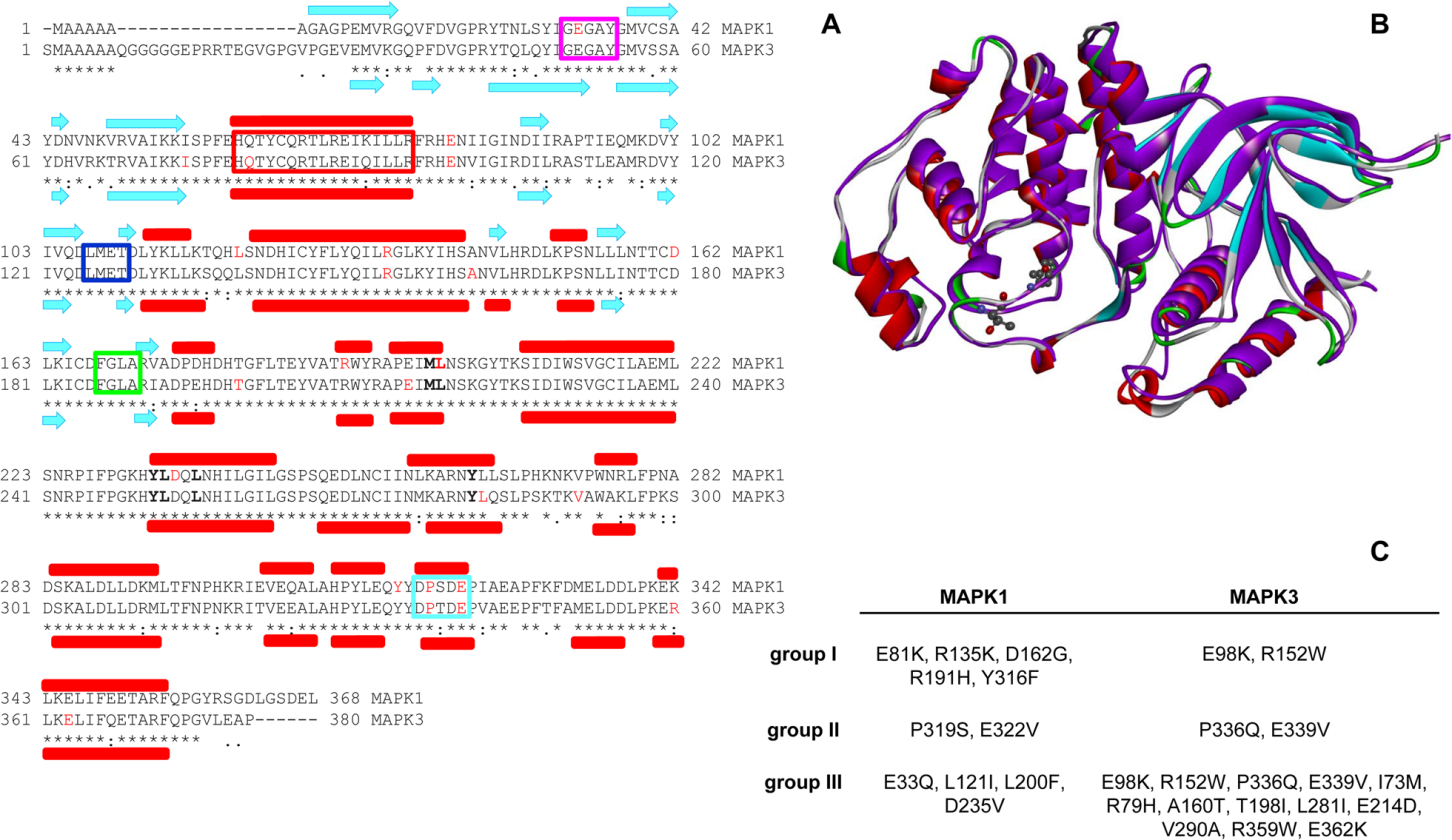
Sequence alignment, structure and variants of MAPK1 and MAPK3. **a** Secondary structure elements (alpha helices in red rectangles, beta-sheet in cyan arrows) are shown at the top (MAPK1) and at the bottom (MAPK3) of the sequence alignment. Mutated residues in MAPK1 and MAPK3 variants are highlighted in red. F-site residues are shown in bold. Pink box: Gly-rich loop; red box: αC-helix; blue box: hinge region; green box: activation loop; CD-site: cyan box. Asterisks indicate the fully conserved residues. **b** Superposition of human MAPK1 (in magenta, pdb: 1TVO), and MAPK3 wild type (secondary structure color, pdb: 4QTB). The residues T185 and Y187 (MAPK1), and T202 and Y204 (MAPK3) important for the protein activation are represented in ball and stick. **c** MAPK1 and MAPK3 variants object of these studies

**Fig. 2:**
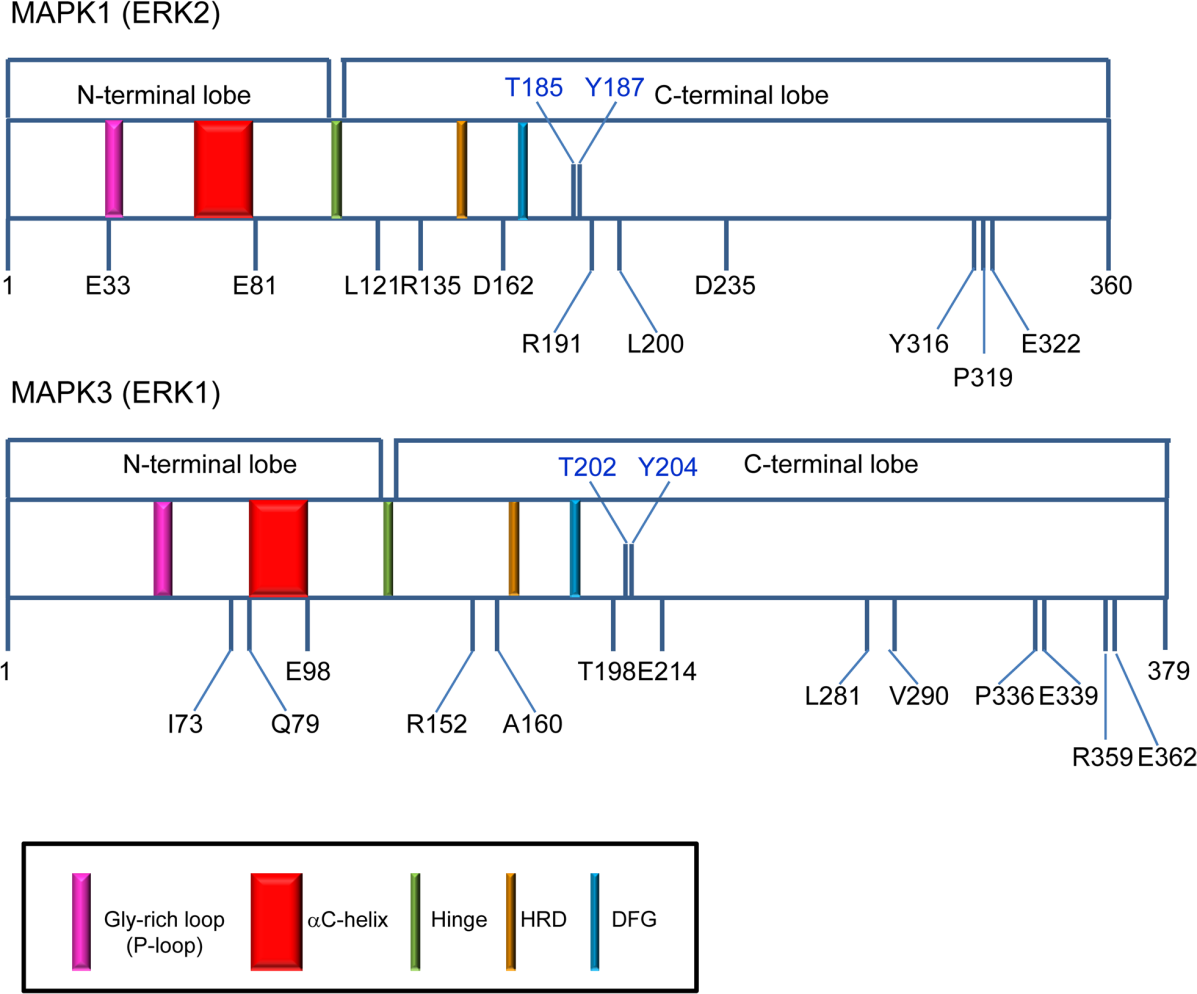
Schematic representation of MAPK1 and MAPK3 domain architecture. The N-terminal lobe contains the P-loop (pink) and the αC-helix (red); the C-terminal lobe contains the catalytic HRD (orange), the conserved DFG sequence (cyan) and the residues T185 and Y187 (MAPK1), and T202 and Y204 (MAPK3) important for the protein activation and catalysis. The N-terminal lobe and the C-terminal lobe are connected by a hinge (green). All the residues mutated in the variants object of this study are indicated

MAPK1 and MAPK3 share more than 80% sequence identity (and 88% similarity) (Fig. 1), as well as the same domain architecture (Fig. 2); however, their physiological redundant role is still controversial [3, 42]. Recent studies suggest that MAPK1 and MAPK3 are functionally interchangeable in both the G1-S transition and in the G2/M checkpoint activation upon DNA damage, other studies point to a differential role for the two isoforms [7, 9]. Interestingly, hydrogen/deuterium experiments on MAPK1 and MAPK3 highlighted a different conformational mobility that controls their enzymatic function upon activation, although the 3D structures of the two isoforms are closely similar [31]. Notably, the MAPK1 and MAPK3 are both quite stable in vivo, with half-life of 68 and 53 h, respectively [3]. In addition, the MAPK1 and 3 show a dramatic difference in the crossing capability of the nuclear envelope [22]. The two kinases show different sensitivity to turnover induced by binding of MAPK inhibitors, and MAPK3 is more resistant than MAPK1 [1]. The two proteins are activated upon phosphorylation of T185 and Y187 for MAPK1 and phosphorylation of T202 and Y204 for MAPK3 (Fig. 2). This event triggers in both the enzymes a significant conformational change which occurs in a cleft between the small N-terminal and the large C-terminal lobe that are connected by a hinge region (Fig. 2). Catalysis requires ATP binding, substrate recognition and binding that encompasses both the N- and C-terminal lobes. The adenine moiety of ATP interacts with the beta strands of the N-terminal lobe, and the interaction with the phosphate group of ATP is stabilized by a conserved glycine-rich loop (also called P-loop) (Figs. 1 and 2). The residues important for substrate binding and catalysis, such as the conserved HRD and DFG sequences and the threonine and tyrosine residues that are phosphorylated in the active conformation, are in the C-terminal lobe (Fig. 2) [32]. The two lobes in the inactive conformation are more open and, upon phosphorylation, become closer in the active conformation [32]. An important change occurs in the N-terminal lobe for the alpha C-helix, which forms part of the active site, by rotating and moving from the rest of the lobe. These structural rearrangements are accompanied by the formation of a salt bridge between MAPK1/3 alpha C-helix E71/E88 and the conserved K54/K71 in the strand beta 3 (Additional file 1: Fig. S1). The aspartate side chain of MAPK1/3 D167/D184 that belongs to the conserved DFG sequence points out from the active site in the inactive conformation, and upon activation, points to the ATP-binding pocket and coordinates Mg^++^, a typical change of the active conformation. The recruitment of MAPK1/MAPK3 substrates and regulators occurs through the F-site and the CD-sites, two docking sites located close to the catalytic cleft and at the opposite of the catalytic cleft, respectively (Fig. 1) [32].

A complex disease like cancer is characterized by dynamic changes in the genome that may confer new and diverse growth advantage and drive the evolution from normal cells into cancer cells [12, 13]. At the basis of the genetic changes there is inflammation, promoted by tumor and genomic instability [39], that is caused by the alteration in the processes that control cell division and that is common in many types of cancer. The genomic changes that may vary from base pair mutations to chromosome instability may contribute to the development of cancer. In the last decades, the cancer genome landscape has been extensively analyzed to distinguish in the tumor samples benign or neutral mutations from cancer-related mutations. A genome-wide association study (GWAS) of solid cancer tissues indicates that in 33–66 genes are found somatic mutations and 95% of the total are represented by single nucleotide variants. These mutations are mainly missense or non-synonymous, lead to a change in the protein sequence [47] and may affect protein conformation, function and/or stability, and they may also alter the interaction with other proteins and ligands.

COSMIC database reports many somatic missense variants of MAPK1 and MAPK3, found in cancer tissues, [43] carrying single mutations in different regions of the sequence. Some of these mutations are particularly interesting since they are associated to resistance to MAPK signaling cascade inhibitor [10, 42]. The point mutations may affect protein conformation in solution and stability, as well as protein function and protein–protein interaction. In general, most of the mutations do not alter dramatically the fold of the protein but cause local changes that reflect on the global properties of the protein [8, 28, 29]. The mutations selected in this study from COSMIC database are distributed all over the MAPK1 and MAPK3 sequence (Fig. 1) to monitor their effects on the biochemical and biophysical properties of the two proteins. Eleven MAPK1 and thirteen MAPK3 variants were selected from COSMIC database to surf the primary structure of the two proteins and are reported in Additional file 1: Table S1.

The thermodynamic stability and the biophysical properties of the wild type and variants have been investigated in the non-phosphorylated (NP-MAPK) and in the phosphorylated (P-MAPK) form. The catalytic properties of the P-MAPK1/3 variants have been studied in vitro on pure recombinant proteins and compared to those of the P-wild type. The detailed descriptions of the changes occurring in the variants are required to understand the consequence of the mutation observed in the MAPK1/3 from cancer tissues since information about the effect of mutations on the biochemical and structural properties of MAPK1/3 is lacking, and most of the studies reported in the literature have not been performed on the pure proteins [42].

## Materials and methods

### Site‐directed mutagenesis

For Escherichia coli expression, pNIC28-Bsa4 plasmid harboring the MAPK1 or MAPK3 wild-type gene was used. The point mutations were introduced on wild‐type gene using QuikChange Lightning Site-Directed Mutagenesis (Agilent Technologies, Santa Clara, CA). Sequence analysis was performed to confirm the presence of the desired mutations and the absence of unwanted ones.

### Protein expression and purification

N-terminally His-tagged MAPK1 and MAPK3 wild type and variants were expressed with phosphatase in E. coli cells Rosetta. *E. coli* cells were grown in LB medium containing kanamycin (30 µg/mL final concentration) at 37 °C until OD_600_ nm reached 0.6 AU. Protein expression was induced by adding 0.5 mM isopropyl‐β‐d‐thiogalactoside (Sigma‐Aldrich, St. Louis, MO), The culture was incubated overnight at 20 °C under shaking and centrifuged. The sedimented material was reconstituted in 40 mL of a solution consisting of 50 mM Hepes, 500 mM NaCl, 5 mM Imidazole, 5% glycerol (pH 7.5), referred to as the “Binding buffer.” This buffer also contained 0.5 mM Tris(2‐carboxyethyl) phosphine and a mixture of protease inhibitors without ethylenediaminetetraacetic acid (EDTA), which were obtained from Sigma‐Aldrich. The cells were subjected to sonication while kept on ice using a Vibracell 75,115 sonicator (SONICS, Newtown, CT) with a cycle of 3 s of sonication followed by 9 s of pause. After sonication, the lysate was subjected to centrifugation. Subsequently, the resulting supernatant was loaded into a Ni–NTA (Ni2 + nitrilotriacetate) affinity column pre-conditioned with Binding buffer. The column was sourced from GE Healthcare in Chicago, IL. The recombinant protein was eluted with 250 mM imidazole in Binding buffer, concentrated to a final volume of 2.5 mL (Amicon concentrator Ultra‐15, Millipore, Burlington, MA) and then applied to a PD‐10 prepacked column (GE Healthcare) to remove the imidazole. The hexahistidine tag was removed by incubation with His‐tag tobacco etch virus (TEV) protease overnight, at 4 °C. The mixture, which included the cleaved protein, the His-tag, and the TEV protease, was loaded into a Ni–NTA affinity column pre-conditioned with the Binding buffer. The protein without His‐tag was present in the flow through.

Protein concentration was determined using a molar absorptivity at 280 nm of 44,810 M^−1^ cm^−1^ referred to a 41.477 kDa molecular mass for MAPK1 wild type and variants, and a molar absorptivity of 43,320 M^−1^ cm^−1^ referred to a 43.222 kDa molecular mass, for MAPK3 wild type and variants. The protein purity was checked by sodium dodecyl sulfate–polyacrylamide gel electrophoresis (SDS‐PAGE) using precast NuPage 4–12% Bis‐Tris polyacrylamide gels (Thermo Fisher). Western blot analyses were performed on the wild type and the variants to assess the presence of the phosphorylated (P-MAPK1 and P-MAPK3) and non-phosphorylated (NP-MAPK1 and NP-MAPK3) form of the protein. The presence of both forms was confirmed through immunodetection using specific antibodies: the anti-ERK1/ERK2 (Thermo Fisher Cat. 44-654G) and the antibody directed against the doubly phosphorylated MAPK3 and MAPK1 (anti-phospho-ERK1/ERK2-Thr185, Tyr187-Thermo Fisher Cat. 44-680G). To activate both MAPK1 and MAPK3, including their wild-type and variant forms, the plasmid pGEX-KG-MEKR4F (an active mutant of MEK1 kindly provided by Prof. Melanie Cobb, Southwestern University, TX) was employed for co-expression of these proteins. The wild-type and variant forms of MAPK1 and MAPK3 were expressed as N-terminally His-tagged proteins within E. coli cells BL21(DE3)-pLysS and subsequently purified with the previously described method.

### Spectroscopic measurements

The intrinsic fluorescence emission spectra (ranging from 300 to 450 nm) for both wild-type and variant forms of MAPK1 and MAPK3 were measured at a temperature of 20 °C. This was done using an LS50B spectrofluorometer (Perkin-Elmer) with protein concentrations in the range of 100–130 μg/mL. The samples were prepared in a solution containing 20 mM Tris/HCl at pH 7.5, 0.1 M NaCl and 200 μM DTT; a quartz cuvette with a path length of 1.0 cm was used.

Circular dichroism (CD) spectra for MAPK1 and MAPK3, both wild type and variants, were recorded in two regions. Far-UV CD spectra (covering the range of 190–250 nm) were obtained at protein concentrations between 100 and 130 μg/mL (equivalent to 0.13 AU at 280 nm). The protein samples were dissolved in 20 mM Tris/HCl at pH 7.5, 0.1 M NaCl and 200 μM DTT. A quartz cuvette with a path length of 0.1 cm was used.

Near-UV CD spectra (spanning 240–420 nm) were monitored with protein concentrations ranging from 1.0 to 1.3 mg/mL (equivalent to 1.3 AU at 280 nm). The buffer composition for these samples was 20 mM Tris/HCl at pH 7.5, 0.1 M NaCl, and 1.0 mM DTT, and a quartz cuvette with a path length of 1.0 cm was utilized. The CD measurements were taken using a Jasco-815 spectropolarimeter (Jasco, Easton, MD, USA), and the results were expressed as [Θ], representing the mean residue ellipticity. This calculation assumed a mean residue molecular mass of 110.

### GdmCl-induced equilibrium unfolding

Wild-type and variant forms of MAPK1 and MAPK3 (final concentration of 75.0 μg/mL) were incubated at increasing concentrations of GdmCl (ranging from 0 to 8 M) at a temperature of 4 °C. This process was conducted in a solution consisting of 20 mM Tris/HCl at pH 7.5, 0.1 M NaCl and 0.2 mM DTT. After a 30-min incubation period to reach equilibrium, intrinsic fluorescence emission and far-UV CD spectra were collected at a temperature of 10 °C using a cuvette with a path length of 0.2 cm. To assess the reversibility of the unfolding, MAPK1 and MAPK3 wild type and variants at a final concentration of 0.75 mg/mL were unfolded in the presence of 8.0 M GdmCl, at a temperature of 10 °C, within a buffer consisting of 20 mM Tris/HCl at pH 7.5, 2 mM DTT and 0.1 M NaCl. After 5 min, the refolding process was initiated by diluting the unfolding mixture tenfold, at 4 °C, into solutions with progressively lower GdmCl concentrations. The unfolding experiments were performed in triplicate.

### Thermal denaturation experiments

Thermal denaturation experiments were carried out by heating MAPK1 and MAPK3 wild type and mutants (100–130 μg/mL) in a 0.1-cm quartz cuvette, from 20 to 90 °C, in 20 mM Tris/HCl, pH 7.5, 0.2 mM DTT, 0.1 M NaCl at a heating rate of 1° × min^−1^ using a Jasco programmable Peltier element. The dichroic activity at 222 nm, along with the photomultiplier signal, was measured [2]. The contribution from the solvent for all thermal scans was monitored at increasing temperatures. Melting temperatures (*T*_m_) were determined analyzing the first derivative of the ellipticity at 222 nm in relation to temperature, as explained in [27]. Each of these measurements was conducted in triplicate.

### Enzyme activity assay and kinetic studies

The enzymatic activity of the purified P-MAPK1 and P-MAPK3 wild type and variants was measured by using Chelation-Enhanced Fluorescence (ChEF) method monitoring the incorporation of phosphate into a substrate peptide [41]. The activity of P-MAPK1 and P-MAPK3, wild-type and variant forms, was measured at a temperature of 30 °C using PhosphoSensR Peptide AQT0490 (provided by AssayQuant Technologies Inc., Marlboro, MA, USA) as the substrate, following the method described in [24]. The reaction mixture consisted of 50 mM Hepes at pH 7.5, 10 mM MgCl_2_, 0.1 M DTT, 0.012% Brij-35, 1% glycerol, 0.2 mg/mL BSA, 5.0 mM MgATP and AQT0490 peptide at concentrations ranging from 0.069 to 40 µM. The final volume of the reaction mixture was 0.4 mL. The reaction was initiated by adding various amounts (0.008–24 μg) of P-MAPK1 or P-MAPK3 wild-type and variant forms. These enzymes were diluted in a solution containing 20 mM Hepes at pH 7.5, 0.01% Brij-35, 0.1 mM EGTA, 5% glycerol, 1 mM DTT and 1 mg/mL BSA. The final enzyme concentrations ranged from 0.5 to 2381 nM for MAPK1 wild type and variants and from 8.0 to 90 nM for MAPK3 wild-type and variants. The increase in fluorescence intensity at 490 nm (with an excitation wavelength at 360 nm), which corresponds to the phosphorylation of the AQT0490 peptide by P-MAPK1 or P-MAPK3, was continuously monitored for a duration of 5 min using an LS50B spectrofluorometer (Perkin-Elmer). The kinetic data were subsequently analyzed using GraphPad Prism 7.03 (La Jolla, CA, USA). The reported results represent the mean values obtained from three separate experiments utilizing distinct enzyme preparations.

### Temperature dependence of P-MAPK1 and P-MAPK3 activity

The temperature dependence of P-MAPK1 and P-MAPK3 catalytic activity was studied by measuring the enzyme activity as a function of temperature. The choice of substrate peptide concentrations for both wild-type and variant forms was based on their respective *K*_m_ values and was consistently set below the *K*_m_ value. To initiate the reaction, 2 μL of pure enzyme (maintained at 10 °C) was added with continuous stirring to a 0.4 mL assay mixture. This assay mixture contained 50 mM Hepes at pH 7.5, 10 mM MgCl_2_, 0.1 M DTT, 0.012% Brij-35, 1% glycerol, 0.2 mg/mL BSA, 5.0 mM MgATP and 1 µM AQT0490 (as explained in the enzyme activity assay section). The entire mixture was equilibrated at different temperatures (10, 15, 20, 25, 30, 35, 37, 40, 42 and 45 °C) within a temperature-controlled cuvette. The final enzyme concentration ranged from 0.5 to 1400 nM. The fluorescence intensity at 490 nm was continuously measured over 5 min. The activation energies (*E*_a_) for the catalytic reaction were obtained by nonlinear fitting to the Arrhenius equation calculating the variation of enzyme activity as a function of temperature

1 $$k = Ae^{{ - E_{{\text{a}}} /RT}}$$

where *k* (s^−1^) is the rate constant at temperature *T* (K), A is a reaction specific quantity, *R* the gas constant (1.987 cal × mol^−1^ × K^−1^), and *E*a is the activation energy of the reaction.

### Data analysis

The variations in intrinsic fluorescence emission spectra caused by GdmCl were measured in terms of the intensity-averaged emission wavelength, $\overline{{\uplambda }}$, [36] calculated according to

2 $$\overline{\lambda } = \sum (Ii/\lambda i)/\sum (Ii)$$

where *λ*_*i*_ represents the emission wavelength, and *I*_*i*_ corresponds to the fluorescence intensity at that specific emission wavelength. The value $\overline{{\uplambda }}$ is an integral measurement, which remains largely unaffected by noise and represents an indicator of alterations in the shape and position of the emission spectrum.

The equilibrium unfolding transitions induced by GdmCl, which were followed using changes in far-UV CD ellipticity or intrinsic fluorescence, were examined by fitting the data from both the baseline and the transition region to a two-state linear extrapolation model [38]. In detail, the data were fitted to the following equation:

3 $$\Delta G_{{{\text{unfolding}}}} = \, \Delta G^{{{\text{H}}_{2} {\text{O}}}} + m\left[ {{\text{GdmCl}}} \right] - {\text{RT}}\,{\text{ln}}\,\left( {K_{{{\text{unfolding}}}} } \right)$$

In this equation, Δ*G*_unfolding_ represents the change in free energy associated with unfolding at a specific GdmCl concentration, $\Delta G^{{{\text{H}}_{{2}} {\text{O}}}}$ is the change in free energy related to unfolding when GdmCl is absent, and *m* denotes the slope term, which quantifies how the unfolding constant (*K*_unfolding_) changes with each unit increase in GdmCl concentration. *R* and *T* are the gas constant and the temperature, respectively, and *K*_unfolding_ is the equilibrium constant for the unfolding process. The model calculates the signal as a function of the GdmCl concentration:

4 $$y_{i} = \frac{{y_{\rm N} + s_{\rm N} [X]_{i} + \left( {y_{\rm U} + s_{\rm U} [X]_{i} } \right)*\exp \left[ {\left( { - \Delta G^{{{\text{H}}_{2} {\text{O}}}} - m[X]_{i} } \right)/RT} \right]}}{{1 + \exp \left[ {\frac{{\left( { - \Delta G^{{{\text{H}}_{2} {\text{O}}}} - m[X]_{i} } \right)}}{RT}} \right]}}$$

where *y*_i_ represents the observed signal, *y*_*U*_ and *y*_*N*_ denote the baseline intercepts for the unfolded and native protein, *s*_*U*_ and *s*_*N*_ signify the baseline slopes for the unfolded and native proteins, [*X*]_*i*_ indicates the GdmCl concentration after the *i*th addition, $\Delta G^{{{\text{H}}_{{2}} {\text{O}}}}$ is the extrapolated unfolding free energy change in the absence of denaturant, and *m* represents the slope of a plot of Δ*G*_unfolding_ versus [*X*].

The data were subjected to a global fitting procedure, with the *m* values shared among the datasets. No constraints were imposed on the other parameters. In accordance with Eq. (3), the denaturant concentration corresponding to the midpoint of the transition, denoted as [GdmCl]_0.5_, was determined as follows:

5 $$\left[ {{\text{GdmCl}}} \right]_{0.5} = \Delta G^{{{\text{H}}_{2} {\text{O}}}} /{\text{m}}$$

GraphPad Prism 7.03 was used to fit all unfolding transition data.

Far-UV CD spectra obtained incrementing the GdmCl concentration were analyzed using a singular value decomposition algorithm (SVD). This analysis, performed by MATLAB software (Math-Works, South Natick, MA), aims to remove from the data the high-frequency noise and low-frequency random errors and to determine the number of independent components within a set of spectra, as outlined in [27].

For all the MAPK3 variants and certain MAPK1 variants, the GdmCl-induced equilibrium unfolding exhibited a non-two-state pattern due to the formation of an intermediate state at low denaturant concentrations. When an intermediate state was observed, the variations in [Θ]_222_ or intrinsic fluorescence resulting from the increasing GdmCl concentrations were fitted to the equation describing a three-state folding process:

6 $$F = \frac{{FN + \exp \left( {m{\text{IN}}\frac{{\left[ {{\text{GdmCl}}} \right] - D50{\text{IN}}}}{RT}} \right) \cdot \left( {FI + FU\exp \left( {m{\text{UI}}\frac{{\left[ {{\text{GdmCl}}} \right] - D50{\text{UI}}}}{RT}} \right)} \right)}}{{1 + \exp \left( {m{\text{IN}}\frac{{\left[ {{\text{GdmCl}}} \right] - D50{\text{IN}}}}{RT}} \right) \cdot \left( {1 + \exp \left( {m{\text{UI}}\frac{{\left[ {{\text{GdmCl}}} \right] - D50{\text{UI}}}}{RT}} \right)} \right)}}$$

where *F* is $\overline{{\uplambda }}$ in Eq. (2), or [Θ]_222_, *m* represents the change in the solvent-accessible surface area involved in the transition. Specifically, D50_I–N_ and *m*_I–N_ denote the midpoint and *m* value for the transition from the native state (N) to the intermediate state (I), while D50_U–I_ and *m*_U–I_ represent the midpoint and *m* value for the transition from the intermediate state (I) to the unfolded state (U) [35]. F_I_ which represents $\overline{{\uplambda }}$ or the [Θ]_222_ at the intermediate state (I), is a constant. *F*_N_ and *F*_U_, which represent the $\overline{{\uplambda }}$ or the [Θ]_222_ of the N and the U state, respectively, show a linear dependence on GdmCl concentration:

7 $$F_{{\text{N}}} = a_{{\text{N}}} + b_{{\text{N}}} \left[ {{\text{GdmCl}}} \right]$$

8 $$F_{{\text{U}}} = \, a_{{\text{U}}} + \, b_{{\text{U}}} \left[ {{\text{GdmCl}}} \right]$$

In this equation, *a*_N_ and *a*_U_ represent the baseline intercepts for N and U, while *b*_N_ and *b*_U_ correspond to the baseline slopes for N and U, respectively. All the data related to unfolding transitions were fitted using GraphPad Prism 7.03.

### Western blot

Following the SDS-PAGE procedure described earlier, the protein bands were transferred onto Hi-Bond N + membranes (Cytiva) using a XCell II Blot Module (Thermo Fisher) with a constant voltage setting of 30 V and 250 mA for 60 min. After blotting, the membranes underwent three washes with Tris-buffered saline (TBS) containing 0.1% Tween^®^ 20 detergent (TBST). Subsequently, the membranes were blocked with TBST containing 5% Bovine Serum Albumin (BSA, Sigma-Aldrich). Following this, the membranes were washed once with TBST and twice with TBS before being exposed to the primary Antibody 44-680G, which is an anti-phospho-ERK1/ERK2 (T185, T187) antibody from Thermo Fisher, raised against the doubly phosphorylated ERK2. The primary antibody was diluted to a 1:5000 concentration and incubated overnight at 25 °C.

Afterward, the membranes, which now had the primary antibody bound to them, were incubated with anti-Rabbit IgG (Thermo Fisher, Catalog # 32,460), diluted to a 1:5.000 concentration in TBS, for 60 min at 25 °C. They were then subjected to three additional washes in TBST. Labeled protein bands were detected using a chemiluminescent system according to the supplier's protocol (Amersham).

The same western blot membranes were subsequently used for re-probing with an antibody specific to the non-phosphorylated form of ERK2, labeled as antibody anti-ERK1/ERK2 (Thermo Fisher, Catalog# 44-654G), after implementing the stripping protocol outlined in [24].

### Molecular dynamics

The crystal structures of the wild-type MAPK1 and MAPK3 were acquired from the Protein Data Bank, representing both the phosphorylated/active state (P-MAPK1: 5v60, P-MAPK3: 2zoq) and the inactive/non-phosphorylated state (MAPK1: 4zzn, MAPK3: 4qtb). To investigate the impact of specific mutations, we generated nine variants for both NP-MAPK1 and P-MAPK1 using in-silico point mutation with PyMOL (The PyMOL Molecular Graphics System, Version 1.2r3pre, Schrödinger, LLC). The variants included E33Q, E81K, R135K, D162G, D321G, D321N, E322K and E322V. Additionally, we examined a variant of MAPK3, namely E98K, which corresponds to the E81K variant observed in MAPK1.

To investigate the dynamic behavior of all twenty-four distinct structures, including both the wild-type proteins and the variants (both phosphorylated and non-phosphorylated), classical molecular dynamics (MD) simulations were conducted using the GROMACS package [11, 14] with the GROMOS43A1-P force field [21]. The MD simulations allowed us to observe and analyze the conformational changes and interactions within the protein structures over time, providing valuable insights into their functional characteristics and stability.

MD simulations were carried out in the NPT ensemble at 315 K and neutral *p*H by following the here reported procedure. The temperature is held fixed at 315 K using the v-rescale thermostat [4] with a coupling time of 0.1 ps. The pressure is kept constant at the reference pressure of 1 bar with a coupling time of 1 ps and an isothermal compressibility of 4.5·10^−5^ bar^−1^, exploiting the features of the Parrinello-Rahman barostat [26]. The simple point charge model is used for water molecules. The simulation box is cubic (with a side of 9.53 nm), and each protein is immersed in an appropriate number of water molecules (in order to have the liquid water density), and Na^+^ and Cl^−^ counterions at a concentration of 0.15 M are added to have a wholly neutral system. Periodic boundary conditions are used throughout the simulation, and the particle mesh Ewald algorithm is used to deal with the long-range Coulomb interactions [6]. A time step of 2 fs was used. A non-bond pair list cutoff of 1.0 nm was used, and the pair list was updated every ten steps. Each protein structure is initially relaxed in vacuum via a steepest descent minimization. Then, the appropriate amount of counterions and water is added. At this point, the whole system, solute and solvent, is relaxed again via a steepest descent minimization and then equilibrated for 100 ps in the NVT ensemble at three increasing temperatures: 150 K, 293 K and 315 K. This is the situation from which the final 800-ns-long NPT MD simulation (for each system) is started.

## Results

### Spectroscopic characterization

**Fig. 3:**
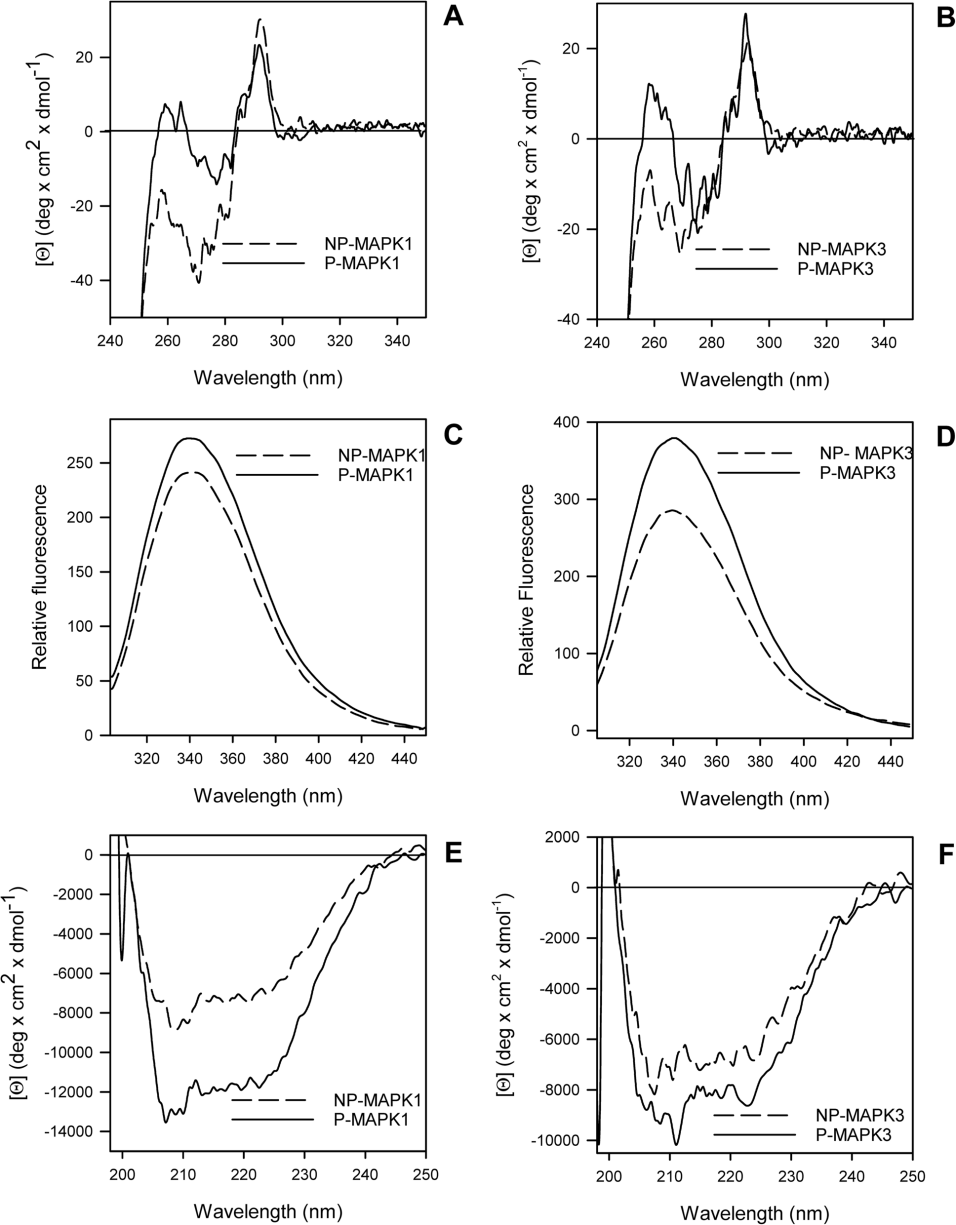
Spectral properties of non-phosphorylated (NP-) and phosphorylated (P-) MAPK1 (**a**, **c**, **e**) and MAPK3 (**b**, **d**, **f**) wild type. **a**, **b** Near-UV CD spectra were recorded in a 1.0-cm quartz cuvette at 1.3 mg/mL protein concentration in 20 mM Tris–HCl pH 7.5 containing 1.0 mM DTT and 0.1 M NaCl. **c**, **d** Intrinsic fluorescence emission spectra (295 nm excitation wavelength) were monitored at 130 µg/mL (0.08 AU 280 nm) in 20 mM Tris–HCl, pH 7.5, 0.1 M NaCl, 0.2 mM DTT. (E, F) Far-UV CD spectra were monitored in a 0.1-cm quartz cuvette at 130–170 µg/mL in 20 mM Tris–HCl, pH 7.5, 0.2 M NaCl and 0.2 mM DTT. The dashed lines are used for the NP-MAPK1 and N-PMAPK3; the continuous lines are used for P-MAPK1 and P-MAPK3. All spectra were recorded at 20 °C

The structural properties of MAPK1/3 wild type and variants obtained in this study as pure recombinant proteins were investigated in solution by circular dichroism (CD) and intrinsic fluorescence spectroscopy in the non-phosphorylated (NP-) and in the phosphorylated (P-) form (Fig. 3). Upon phosphorylation the near-UV CD spectrum of wild-type MAPK3 at around 260–270 nm region, and the intrinsic fluorescence spectrum show changes similar to what observed for MAPK1, i.e., a change in intrinsic fluorescence intensity with no change in maximum emission wavelength, in line with the near-UV CD spectrum showing no changes in tryptophan environment [24], suggesting that tertiary rearrangements follow the active site phosphorylation also for MAPK3 (Fig. 3).

The selected variants of MAPK1/3 have been classified in three different groups, based upon the mutated residue interaction, or not, with the common docking site (CD-site) (Fig. 1). CD-site is a protein energetic hot spot, constituted by two negatively charged residues and of a hydrophobic component, which interacts with proteins as substrate and as regulators and that is involved in the MAPK1/3 protein functions. MAPK1 group I variants, mutated residue interacting with the CD-site: E81K, R135K, D162G, R191H, Y316F; group II variants, mutated residue in the CD-site: P319S and E322V; group III variants, mutated residue in other regions of the protein: E33Q, L121I, L200F and D235V.

Similarly, MAPK3 variants have been divided into three groups. Group I variants, mutated residue directly interacting with the CD-site: E98K (corresponding to MAPK1 E81K), R152W (corresponding to MAPK1 R135K); group II variants, mutated residue in the CD-site: P336Q (corresponding to MAPK1 P319S) and E339V (corresponding to MAPK2 E322V); group III variants, mutated residue in other regions of the protein: I73M, Q79H,, A160T, T198I, E214D, L281I, V290A, R359W, E362K. In the MAPK3 variants selection, the mutations are more frequently observed in other region of the proteins in comparison with MAPK1 (Fig. 1).

**Fig. 4:**
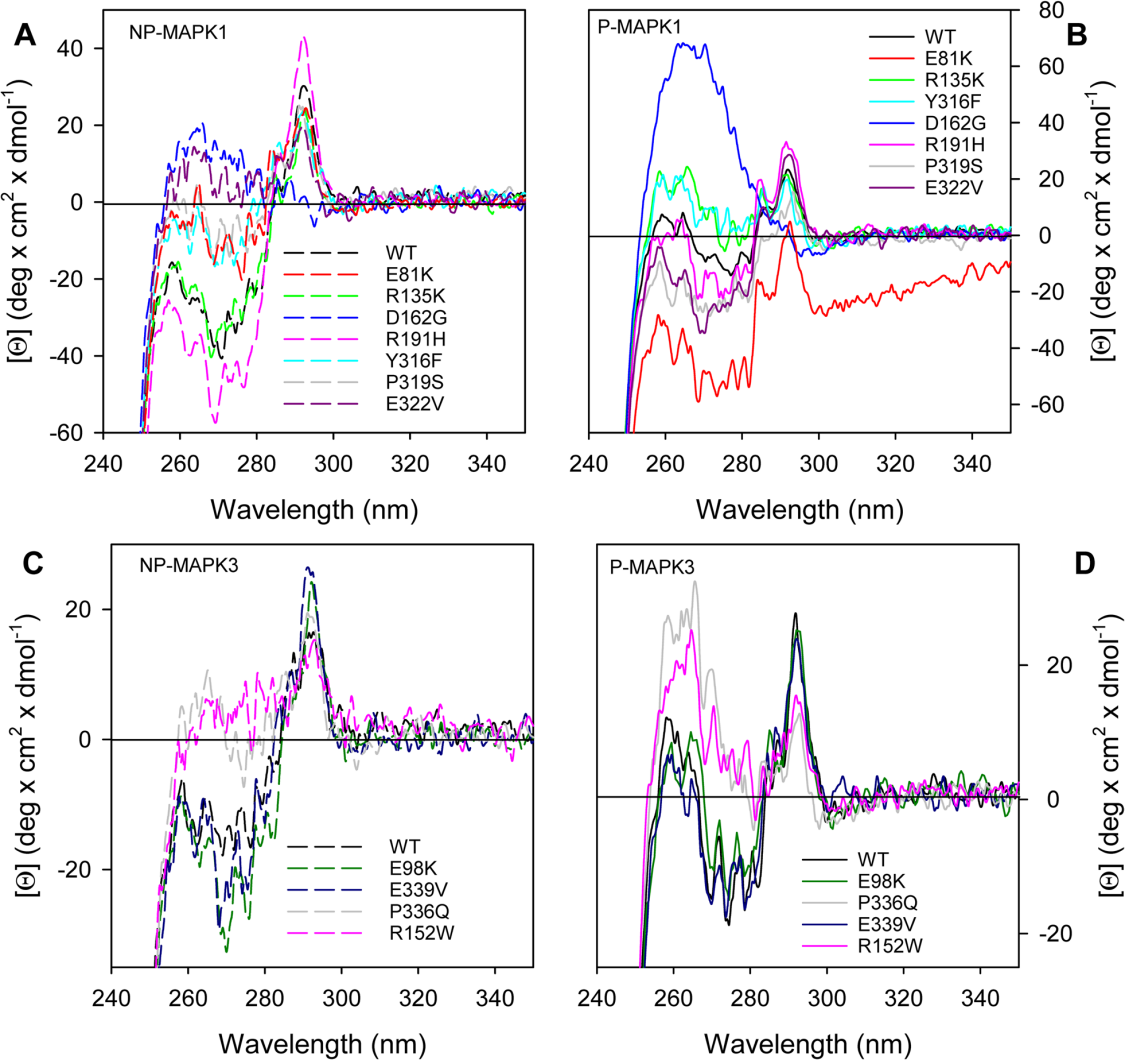
Near-UV CD of non-phosphorylated (NP-) and phosphorylated (P-) MAPK1 and MAPK3 group I and group II variants. Near-UV CD spectra of NP-MAPK1 (A, dashed line), NP-MAPK3 (C, dashed line), P-MAPK1 (B, continuous line) and P-MAPK3 variants (D, continuous line), were monitored at 1.3 mg/mL protein concentration in 20 mM Tris–HCl pH 7.5, 1.0 mM DTT, 0.1 M NaCl, in a 1.0-cm quartz cuvette

**Fig. 5:**
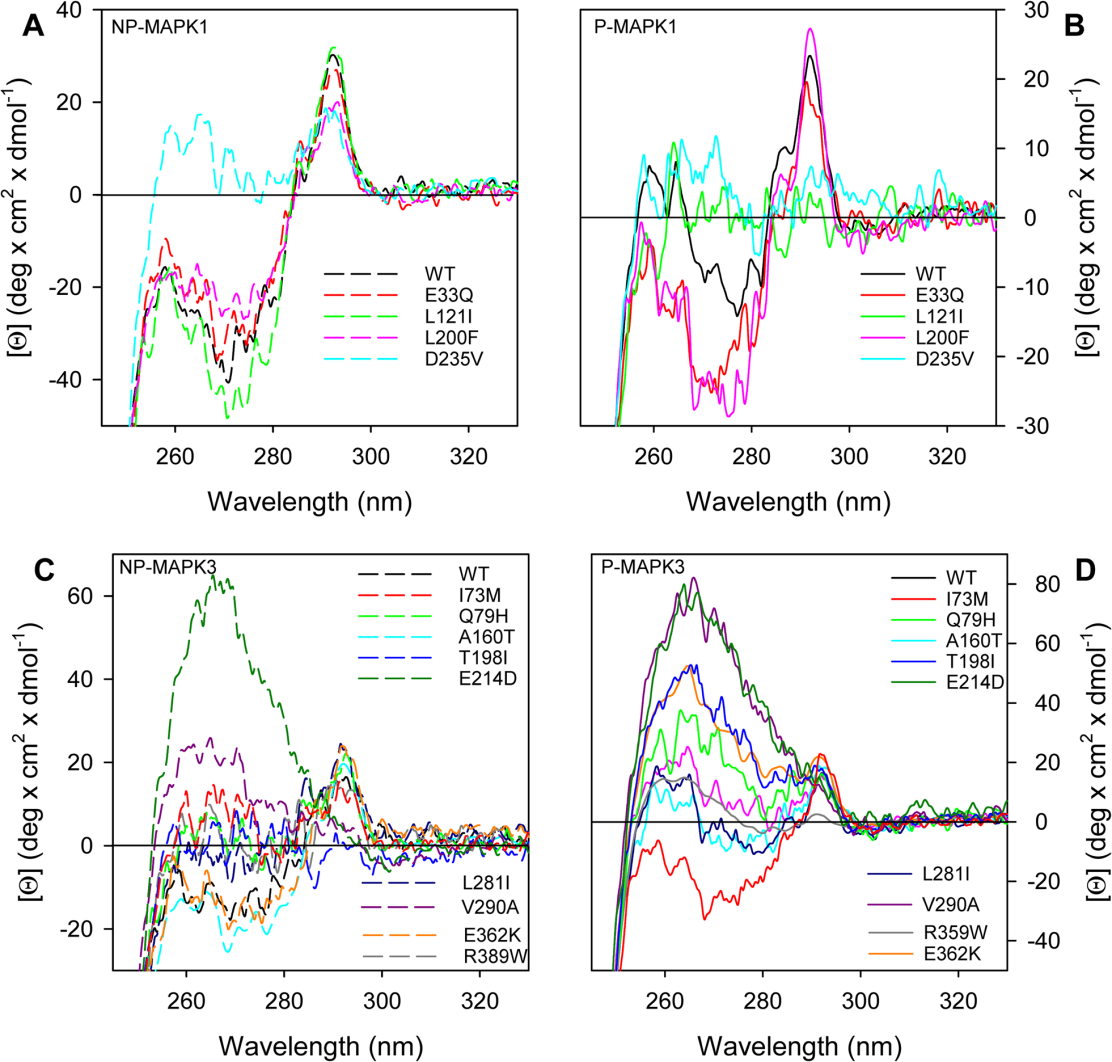
Near-UV CD of non-phosphorylated (NP-) and phosphorylated (P-) MAPK1 and MAPK3 group III variants. Near-UV CD spectra of NP-MAPK1 (**a**, dashed line), NP-MAPK3 (**c**, dashed line), P-MAPK1 (**b**, continuous line) and P-MAPK3 variants (**d**, continuous line), were monitored at 1.3 mg/mL protein concentration in 20 mM Tris–HCl pH 7.5, 1.0 mM DTT, 0.1 M NaCl, in a 1.0-cm quartz cuvette

Comparison of the MAPK1 and MAPK3 near-UV CD spectra (Figs. 4 and 5), indicative of tertiary structure arrangements, of the three groups of variants with that of the corresponding wild type indicates that, independently from their phosphorylation state, MAPK1 variants are all different from the wild type, with the notable exception of R135K (Figs. 4a, b and 5a, b). The changes are mainly localized in the 260–285-nm spectral region (Figs. 4a, b and 5a, b). Only for the P-state of E81K, L121I, D162G and D235V the changes extend in the region around 290 nm typical of tryptophan (Figs. 4 and 5). The variants more different with respect to wild type are D162G and D235V, independently from their phosphorylation state. For MAPK3 group I and II variants (Fig. 4c, d), the tertiary arrangements, as deduced from the near-UV CD spectra, are like that of the wild type, except for P336Q and R152W, which greatly differ from the wild type in both the P- and NP-state (Fig. 4c, d). The tertiary structure of MAPK3 group III variants (Fig. 5c, d) shows significant differences in comparison with the wild type, either in the NP- and P-state, particularly for E214D. Upon phosphorylation, the tertiary structure changes that occur in the wild-type MAPK1 and, to a minor extent, in MAPK3 are localized in the 260–285-nm spectral region (Figs. 3, 4, 5). Among the mutants, all the MAPK1 groups variants are significantly affected, for MAPK3 only P336Q and R152W in group II and the group III mutants show significant changes in the near-UV CD spectra upon phosphorylation.

The intrinsic fluorescence emission spectra of group I, II and III of NP-MAPK1 (Additional file 1: Fig. S2 A, E) and P-MAPK1 (Additional file 1: Fig. S2 B, F) variants differ from that of the wild type only in the intensities. NP-R191H only (Additional file 1: Fig. S2 A, pink dashed line) shows a modest blue-shift of the maximum emission wavelength, probably indicative of a more compact tertiary arrangement, as also suggested by the near-UV CD spectrum where all the aromatic contributions are sharply defined (Fig. 4 A). In group I and II of NP-MAPK3 the spectral differences are modest and occur only in the fluorescence intensities (Additional file 1: Fig. S2 C), a notable quenching in the fluorescence intensity of P-P336Q is observed, upon phosphorylation (Additional file 1: Fig. S2 D, gray continuous line). The largest changes in intrinsic fluorescence among the MAPK3 variants are observed in group III (Additional file 1: Fig. S2 G, H), independently from the phosphorylation state, in parallel to the results observed for the near-UV CD spectra (Fig. 5 C, D).

The far-UV CD spectra (Additional file 1: Fig. S3) that represent the secondary structure elements show minor differences in MAPK1 group I and II upon phosphorylation, much less evident for MAPK3. For both MAPK1 and MAPK3 group III the phosphorylation is followed by far-UV CD spectral changes.

### Thermal and thermodynamic stability studies

The comparison of thermal and thermodynamic stability data, as well as the spectral studies in solution, aims to answer to several questions, (i) is there a difference in the stability of the MAPK1 and 3? (ii) is there a stability change upon wild type MAPK1/3 phosphorylation ? (iii) is there an impact of point mutations on the thermal and thermodynamic stabilities? (iv) is there a stability change upon phosphorylation of the variants?

The thermal stability of both NP- and P-forms of MAPK1/3, including their wild-type versions and variants, was assessed by continuously tracking changes in ellipticity at 222 nm over a temperature range spanning from 20 to 90 °C (as illustrated in Additional file 1: Fig. S4). The monitoring was focused at 222 nm, where the main amplitude was observed. Results indicated an apparent cooperative transition for NP- and P-MAPK1/3 wild-type proteins as well as all the variants. The apparent *T*_m_ values, signifying the midpoint of this transition, fell within a range of 42 to 62 °C (as detailed in Tables 1, 2, 3, 4). It is important to note that the thermal unfolding transitions were found to be irreversible, as evidenced by the signals observed when the protein solutions were cooled from 90 to 20 °C. Interestingly, between the *T*_m_ of MAPK1 and MAPK3 wild type there is a remarkable 9 °C and 8 °C difference NP- and P-state, respectively. Upon phosphorylation no significant difference was observed for P-MAPK1 and a modest 1.1 °C increase for P-MAPK3. As reported in Table 1, T_m_ values of MAPK1 group I and II mutants are like that of the wild type, except for the 5 °C increase observed for NP-and P-D162G and the 4 °C decrease for NP-P319S. In the NP-MAPK3 group I and II, a 6 °C increase in the *T*_m_ of NP-E98K, a 3 °C increase and a 2 °C decrease for NP-R152W and NP-P336Q, respectively, are observed (Table 2). In MAPK1 group III (Table 3) there are 4, 7 and 6 °C increase in the *T*_m_ values of P-E33Q, P-L211I and P-D235V, respectively, with respect to the wild type and in the case of NP-D235V the *T*_m_ is decreased to 52 °C. In the MAPK3 group 3 (Table 4) there are significant differences observed either in comparison with the wild type as well as between the NP- and P-state, as for example in the case of P-V290A and P-R359W. In addition, in this group the thermal transitions are often biphasic and intermediate states become populated during the melting (Table 4).

**Table 1:** Effect of single amino acid substitution on MAPK1 group I and II variants stability^a^

	*T*_m_ (°C)	$\Delta G^{{{\text{H}}_{{2}} {\text{O}}}}$ (kcal/mol)		*m* (kcal/mol/M)		[GdmCl]_0.5_ (M)	
		CD ([$\Theta$]_222_)	Fluorescence ($\overline{{\uplambda }}$)	CD ([$\Theta$]_222_)	Fluorescence ($\overline{{\uplambda }}$)	CD ([$\Theta$]_222_)	Fluorescence ($\overline{{\uplambda }}$)
NP-wild type	55.0	2.51 ± 0.22	2.79 ± 0.16	0.91 ± 0.09	1.34 ± 0.08	2.76	2.08
P-wild type	55.0	2.30 ± 0.16	2.88 ± 0.20	0.97 ± 0.07	1.19 ± 0.09	2.37	2.42
NP-E81K	55.0	1.10 9.03	2.76 7.02	1.77 ± 0.18 2.98 ± 0.40	2.07 ± 0.24 2.26 ± 0.70	0.62 ± 0.04 3.03 ± 0.08	1.33 ± 0.03 3.10 ± 0.08
P-E81K	54.1	6.04 5.37	5.23 4.72	4.91 ± 0.68 1.49 ± 0.37	4.91 ± 0.37 1.23 ± 0.30	1.23 ± 0.03 3.61 ± 0.14	1.07 ± 0.01 3.84 ± 0.26
NP-R135K	55.1	2.54 6.47	2.77 ± 0.25	2.28 ± 0.19 1.96 ± 0.51	1.32 ± 0.13	1.11 ± 0.02 3.30 ± 0.07	2.10
P-R135K	55.1	2.09 ± 0.17	2.38 ± 0.18	0.99 ± 0.07	1.18 ± 0.09	2.12	2.02
NP-D162G	49.0 60.0	2.28 ± 0.16	2.67 ± 0.12	1.05 ± 0.08	1.00 ± 0.05	2.17	2.68
P-D162G	60.0	3.45 2.53	2.11 ± 0.10	3.46 ± 0.58 1.10 ± 0.12	0.86 ± 0.05	1.00 ± 0.03 2.31 ± 0.05	2.45
NP-R191H	58.0	2.65 6.04	5.29 ± 1.06	2.00 ± 0.27 1.56 ± 0.41	2.76 ± 0.55	1.33 ± 0.04 3.87 ± 0.10	1.92
P-R191H	58.1	2.31 ± 0.19	2.55 ± 0.19	1.02 ± 0.09	1.35 ± 0.11	2.27	1.88
NP-Y316F	55.1	2.54 ± 0.23	2.78 ± 0.25	1.09 ± 0.10	1.39 ± 0.13	2.32	2.00
P-Y316F	55.1	1.46 ± 0.09	2.37 ± 0.19	0.93 ± 0.05	1.09 ± 0.09	1.57	2.17
NP-P319S	51.0	2.23 4.16	3.08 6.44	2.04 ± 0.27 1.17 ± 0.41	2.48 ± 0.23 1.88 ± 0.46	1.09 ± 0.03 3.56 ± 0.20	1.24 ± 0.02 3.43 ± 0.10
P-P319S	55.1	3.04 4.30	2.59 ± 0.24	2.79 ± 0.20 1.12 ± 0.34	1.29 ± 0.13	1.09 ± 0.01 3.84 ± 0.26	2.02
NP-E322V	57.1	4.10 4.14	2.60 ± 0.19	3.63 ± 0.65 1.19 ± 0.32	1.12 ± 0.09	1.13 ± 0.03 3.48 ± 0.15	2.31
P-E322V	56.0	1.89 5.43	2.60 ± 0.22	2.10 ± 0.28 1.60 ± 0.24	1.28 ± 0.11	0.90 ± 0.03 3.39 ± 0.07	2.04

^a^Melting temperatures (T_m_) were obtained as described in Materials and Methods by continuously monitoring the molar ellipticity at 222 nm every 0.5 °C. GdmCl-induced unfolding equilibrium data were measured as described in Materials and Methods by monitoring the ellipticity at 222 nm ([Θ_222_]) and the fluorescence intensity-averaged emission wavelength (λ¯¯¯λ¯, Eq. (2)). ΔG^H^_2_^O^ and *m* values were obtained from Eq. (3); [GdmCl]_0.5_ was calculated from Eq. (5). [Θ_222_] and ‾λ data were fitted to Eq. (4) for the 2-state unfolding or to Eq. (6), for the 3-state unfolding. Data are reported as the mean ± SE of the fit. All the measures were performed on the non-phosphorylated (NP-) and phosphorylated (P-) form of the proteins

**Table 2:** Effect of single amino acid substitution on MAPK3 group I and II variants stability^a^

	*T*_m_ (°C)	$\Delta G^{{{\text{H}}_{{2}} {\text{O}}}}$ (kcal/mol)		*m* (kcal/mol/M)		[GdmCl]_0.5_ (M)	
		CD ([$\Theta$]_222_)	Fluorescence ($\overline{{\uplambda }}$)	CD ([$\Theta$]_222_)	Fluorescence ($\overline{{\uplambda }}$)	CD ([$\Theta$]_222_)	Fluorescence ($\overline{{\uplambda }}$)
NP-WT	46.0	1.84 ± 0.12	2.26 ± 0.14	0.93 ± 0.07	1.11 ± 0.08	1.98	2.04
P-WT	47.1	1.72 ± 0.09	2.40 ± 0.12	0.83 ± 0.04	1.22 ± 0.06	2.07	1.97
NP-E98K	52.0	3.65 2.92	2.92 4.32	4.69 ± 0.88 0.96 ± 0.25	2.86 ± 0.68 1.25 ± 0.56	0.78 ± 0.05 3.03 ± 0.19	1.02 ± 0.04 3.46 ± 0.33
P-E98K	47.1	3.95 3.69	4.66 2.68	3.73 ± 0.29 1.18 ± 0.09	4.48 ± 0.07 0.78 ± 0.01	1.06 ± 0.01 3.12 ± 0.04	1.04 ± 0.02 3.43 ± 0.17
NP-R152W	49.0	2.60 3.84	2.61 ± 0.13	3.55 ± 0.90 0.96 ± 0.60	1.10 ± 0.06	0.73 ± 0.05 3.99 ± 0.71	2.37
P-R152W	42.0 58.0	3.20 3.02	2.12 ± 0.14	4.32 ± 0.84 1.19 ± 0.36	1.00 ± 0.07	0.74 ± 0.06 2.54 ± 0.17	2.11
NP-P336Q	44.0	3.28 3.86	3.52 2.89	4.39 ± 0.88 1.24 ± 0.16	3.75 ± 0.44 0.93 ± 0.08	0.75 ± 0.03 3.11 ± 0.07	0.94 ± 0.02 3.09 ± 0.06
P-P336Q	47.0	2.91 3.08	7.06 3.83	3.03 ± 0.53 1.11 ± 0.16	6.03 ± 0.68 1.57 ± 0.22	0.96 ± 0.03 2.78 ± 0.08	1.17 ± 0.05 2.44 ± 0.05
NP-E339V	46.1	2.83 2.92	5.51 6.43	3.29 ± 0.59 1.06 ± 0.10	5.32 ± 0.75 1.74 ± 0.42	0.86 ± 0.03 2.76 ± 0.05	1.04 ± 0.04 3.70 ± 0.49
P-E339V	48.1	2.80 3.00	7.28 4.19	3.00 ± 0.51 0.94 ± 0.12	6.67 ± 0.12 1.64 ± 0.32	0.94 ± 0.03 3.18 ± 0.08	1.08 ± 0.02 2.55 ± 0.08

^a^Melting temperatures (T_m_) were obtained as described in Materials and Methods by continuously monitoring the molar ellipticity at 222 nm every 0.5 °C. GdmCl-induced unfolding equilibrium data were measured as described in Materials and Methods by monitoring the ellipticity at 222 nm ([Θ_222_]) and the fluorescence intensity-averaged emission wavelength (λ¯¯¯λ¯, Eq. (2)). Δ*G*^H^_2_^O^ and m values were obtained from Eq. (3); [GdmCl]_0.5_ was calculated from Eq. (5). [Θ_222_] and ‾λ data were fitted to Eq. (4) for the 2-state unfolding or to Eq. (6), for the 3-state unfolding. Data are reported as the mean ± SE of the fit. All the measures were performed on the non-phosphorylated (NP-) and phosphorylated (P-) form of the proteins

**Table 3:** Effect of single amino acid substitution on MAPK1 group III variants stability^a^

	*T*_m_ (°C)	$\Delta G^{{{\text{H}}_{{2}} {\text{O}}}}$(kcal/mol)		*m* (kcal/mol/M)		[GdmCl]_0.5_ (M)	
		CD ([$\Theta$]_222_)	Fluorescence ($\overline{{\uplambda }}$)	CD ([$\Theta$]_222_)	Fluorescence ($\overline{{\uplambda }}$)	CD ([$\Theta$]_222_)	Fluorescence ($\overline{{\uplambda }}$)
NP-WT	55.0	2.51 ± 0.22	2.79 ± 0.16	0.91 ± 0.09	1.34 ± 0.08	2.76	2.08
P-WT	55.0	2.30 ± 0.16	2.88 ± 0.20	0.97 ± 0.07	1.19 ± 0.09	2.37	2.42
NP-E33Q	54.1	2.44 ± 0.14	3.33 ± 0.31	0.96 ± 0.06	1.97 ± 0.20	2.54	1.69
P-E33Q	59.0	2.22 7.18	2.98 ± 0.24	1.56 ± 0.10 2.86 ± 0.62	1.30 ± 0.11	1.42 ± 0.03 3.43 ± 0.05	2.29
NP-L121I	55.0	2.29 ± 0.14	3.27 ± 0.31	1.00 ± 0.07	1.41 ± 0.14	2.29	2.32
P-L121I	62.0	1.73 ± 0.11	2.82 ± 0.21	1.12 ± 0.07	1.46 ± 0.12	1.55	1.93
NP-L200F	54.0	2.27 ± 0.16	2.88 ± 0.16	0.96 ± 0.07	1.44 ± 0.08	2.36	2.00
P-L200F	56.1	2.15 ± 0.19	3.06 ± 0.30	0.97 ± 0.09	1.72 ± 0.18	2.20	1.77
NP-D235V	52.1	2.46 ± 0.16	2.48 ± 0.19	1.07 ± 0.08	1.15 ± 0.09	2.30	2.15
P-D235V	61.1	1.77 ± 0.11	2.41 ± 0.23	1.07 ± 0.07	1.10 ± 0.12	1.65	2.18

^a^Melting temperatures (T_m_) were obtained as described in Materials and Methods by continuously monitoring the molar ellipticity at 222 nm every 0.5 °C. GdmCl-induced unfolding equilibrium data were measured as described in Materials and Methods by monitoring the ellipticity at 222 nm ([Θ_222_]) and the fluorescence intensity-averaged emission wavelength (λ¯¯¯λ¯, Eq. (2)). Δ*G*^H^_2_O and m values were obtained from Eq. (3); [GdmCl]_0.5_ was calculated from Eq. (5). [Θ_222_] and ‾λ data were fitted to Eq. (4) for the 2-state unfolding or to Eq. (6), for the 3-state unfolding. Data are reported as the mean ± SE of the fit. All the measures were performed on the non-phosphorylated (NP-) and phosphorylated (P-) form of the proteins

**Table 4:** Effect of single amino acid substitution on MAPK3 group III variants stability^a^

	*T*_m_ (°C)	$\Delta G^{{{\text{H}}_{{2}} {\text{O}}}}$(kcal/mol)		*m* (kcal/mol/M)		[GdmCl]_0.5_ (M)	
		CD ([$\Theta$]_222_)	Fluorescence ($\overline{{\uplambda }}$)	CD ([$\Theta$]_222_)	Fluorescence ($\overline{{\uplambda }}$)	CD ([$\Theta$]_222_)	Fluorescence ($\overline{{\uplambda }}$)
NP-WT	46.0	1.84 ± 0.12	2.26 ± 0.14	0.93 ± 0.07	1.11 ± 0.08	1.98	2.04
P-WT	47.1	1.72 ± 0.09	2.40 ± 0.12	0.83 ± 0.04	1.22 ± 0.06	2.07	1.97
NP-I73M	45.0	2.38 8.74	3.53 4.60	2.71 ± 0.35 2.74 ± 0.42	3.39 ± 0.37 1.46 ± 0.36	0.88 ± 0.02 3.19 ± 0.08	1.04 ± 0.02 3.16 ± 0.18
P-I73M	46.0 58.0	2.31 6.19	2.29 ± 0.16	2.30 ± 0.33 1.81 ± 0.69	1.20 ± 0.09	1.00 ± 0.03 3.43 ± 0.16	1.91
NP-Q79H	44.1 59.0	2.66 2.99	2.28 ± 0.24	3.19 ± 0.94 1.01 ± 0.20	1.14 ± 0.14	0.83 ± 0.05 2.95 ± 0.12	1.99
P-Q79H	47.1	2.91 2.22	2.38 ± 0.20	3.07 ± 0.46 0.85 ± 0.10	1.33 ± 0.13	0.95 ± 0.03 2.60 ± 0.07	1.79
NP-A160T	42.1	5.52 6.41	1.89 ± 0.09	7.53 ± 0.75 1.78 ± 0.24	0.89 ± 0.05	0.73 ± 0.02 3.59 ± 0.06	2.12
P-A160T	44.1	3.22 2.32	3.28 2.54	3.21 ± 0.68 0.86 ± 0.09	4.31 ± 0.47 1.33 ± 0.14	1.00 ± 0.04 2.70 ± 0.06	0.76 ± 0.01 1.90 ± 0.05
NP-T198I	58.0 75.0	1.45 ± 0.11	2.31 ± 0.09	0.99 ± 0.07	1.01 ± 0.42	1.62	2.29
P-T198I	48.0	3.55 7.84	4.08 3.91	4.37 ± 1.02 2.36 ± 0.72	4.52 ± 0.93 1.31 ± 0.11	0.81 ± 0.03 3.31 ± 0.09	0.90 ± 0.03 3.00 ± 0.04
NP-E214D	48.0 59.0 74.0	1.42 2.58	2.81 3.93	2.40 ± 0.55 0.87 ± 0.29	2.38 ± 0.79 1.64 ± 0.15	0.59 ± 0.06 2.96 ± 0.22	1.18 ± 0.09 2.39 ± 0.03
P-E214D	51.0 61.1	3.96 2.60	2.37 3.25	4.03 ± 0.40 1.05 ± 0.07	2.41 ± 0.40 1.69 ± 0.24	0.99 ± 0.01 2.47 ± 0.04	0.99 ± 0.04 1.93 ± 0.05
NP-L281I	46.1	1.94 5.14	4.38 7.72	2.00 ± 0.22 1.31 ± 0.38	3.59 ± 0.36 2.15 ± 0.29	0.97 ± 0.03 3.90 ± 0.16	1.22 ± 0.02 3.59 ± 0.06
P-L281I	47.1	2.40 2.22	4.72 5.53	2.63 ± 0.63 0.84 ± 0.13	4.50 ± 0.33 1.62 ± 0.23	0.91 ± 0.05 2.64 ± 0.10	1.05 ± 0.01 3.41 ± 0.07
NP-V290A	47.0	2.86 5.24	7.52 2.75	3.05 ± 0.35 1.58 ± 0.33	9.19 ± 1.26 1.14 ± 0.11	0.94 ± 0.02 3.31 ± 0.10	0.82 ± 0.02 2.42 ± 0.06
P-V290A	56.0	1.94 2.54	3.20 ± 0.39	1.97 ± 0.46 0.92 ± 0.16	1.22 ± 0.16	0.98 ± 0.06 2.76 ± 0.10	2.62
NP-R359W	43.1	2.85 3.49	2.51 ± 0.14	3.14 ± 0.66 1.05 ± 0.15	1.04 ± 0.06	0.91 ± 0.04 3.33 ± 0.09	2.40
P-R359W	56.1	2.89 3.49	2.76 ± 0.16	3.17 ± 0.67 1.04 ± 0.14	1.22 ± 0.08	0.91 ± 0.04 3.36 ± 0.04	2.26
NP-E362K	44.1	3.69 3.53	4.11 2.02	4.13 ± 0.81 1.13 ± 0.18	3.83 ± 0.55 0.90 ± 0.10	0.89 ± 0.03 3.11 ± 0.09	1.07 ± 0.02 2.23 ± 0.06
P-E362K	46.0	1.26 5.67	3.51 2.54	2.07 ± 0.18 1.62 ± 0.45	3.42 ± 0.75 0.97 ± 0.16	0.61 ± 0.03 3.51 ± 0.13	1.02 ± 0.04 2.61 ± 0.09

^a^Melting temperatures (T_m_) were obtained as described in Materials and Methods by continuously monitoring the molar ellipticity at 222 nm every 0.5 °C. GdmCl-induced unfolding equilibrium data were measured as described in Materials and Methods by monitoring the ellipticity at 222 nm ([Θ_222_]) and the fluorescence intensity-averaged emission wavelength (λ¯¯¯λ¯, Eq. (2)). Δ*G*^H^_2_O and *m* values were obtained from Eq. (3); [GdmCl]_0.5_ was calculated from Eq. (5). [Θ_222_] and ‾λ data were fitted to Eq. (4) for the 2-state unfolding or to Eq. (6), for the 3-state unfolding. Data are reported as the mean ± SE of the fit. All the measures were performed on the non-phosphorylated (NP-) and phosphorylated (P-) form of the proteins

The effect of point mutations on MAPK1/3 thermodynamic stabilities in both NP- and P-states was studied at 10 °C using increasing (0–8 M) [GdmCl] as denaturant and was analyzed in parallel by monitoring the changes in molar ellipticity at 222 nm, using far-UV CD, and the changes in intrinsic fluorescence spectroscopy. The variations in intrinsic fluorescence were quantified through the determination of the intensity-averaged emission wavelength using Eq. (2). The thermodynamic characteristics obtained from the examination of far-UV CD changes and intrinsic fluorescence changes due to the increasing GdmCl concentration are summarized in Tables 1, 2, 3, 4. For most of the variants, the unfolding transitions induced by GdmCl, as observed in far-UV CD, are equivalent with those observed in intrinsic fluorescence. In the case of MAPK1 group I and II (Table 1) three variants out of seven, E81K, P319S and E322V, show a more than two-states unfolding transition in both the NP- and P-state (Additional file 1: Fig. S5). R135K and R191H in the NP-state show a more than two-states unfolding transition and unfold in a cooperative two states in the P-state. Y316F is the only exception and unfolds similarly to the wild type in a two-states cooperative transition in both NP- and P-form. D162G, only in the P-state, unfolds in a more than two-states transitions. In the MAPK1 group III (Table 3) only the P-E33Q shows a more than two-states transition; all the other variants show an apparent two-state unfolding. As far as concern the MAPK3 group I and II (Table 2), all the variants show a more than two-states unfolding transition. A similar trend is observed in MAPK3 group III (Table 4), with the notable exception of NP-T198I that unfolds in a cooperative two-states transition. The population of a denaturation intermediate, suggested by the three-state unfolding transition, could be regarded as a stabilization of the native state because of the increased difference in the unfolding free energy between the native and the unfolded state. Notably both the MAPK1 and MAPK3 wild-type proteins unfold in a two-state cooperative transition in the NP- and P-state with no significant stability change upon phosphorylation; thus, the mutation in the variants stabilizes an unfolding intermediate which becomes populated at equilibrium.

### Functional analysis of the variants

The impact of missense mutations on the kinetic characteristics of MAPK1/3 was assessed by continuously monitoring the rise in fluorescence intensity over time, which resulted from the addition of phosphate to a peptide substrate (PhosphoSensR Peptide AQT0490). This activity assay was conducted under consistent conditions at a temperature of 30 °C, with a constant concentration of MgCl_2_. In group I and II MAPK1 variants (Table 5) the catalytic efficiency (*k*_cat_/*K*_M_) of four out of seven was decreased and for three of them the *k*_cat_/*K*_M_ was reduced from a modest 3- to a notable 1877- and 3352-fold in the case of E81K, D162G and R191H, respectively, whereas for all the other variants was modestly increased, or comparable to that of the wild type. The catalytic efficiency of all MAPK1 group III variants (Table 5) was decreased, ranging from a 2- to the 162-fold decrease measured for L200F (Table 5). In the case of MAPK3 group I, II and III variants (Table 6) the values of the catalytic efficiency are like that of the wild type for A160T, L281I and R359W, moderately increased for T198I and E98K and decreased for all the others from a 2- to a 13-fold, a notably lesser extent in comparison with what observed for MAPK1.

**Table 5:** Kinetic parameters and effect of temperature on kinase activity of MAPK1 variants wild type and variants^a^

	*K*_m_ (μM)	*k*cat (s^−1^)	[enzyme] (nM)	*k*cat/*K*_m_ (s^−1^ μM^−1^)	*V*_max_ (μM/sec)	*T*_max_ (°C)	*E*_a_ (kcal/mol)
Wild type	1.81 ± 0.29	2.20	5.0	1.22	1.10 × 10^–2^	30.0	9.95 ± 0.41
*GROUP I and II*							
E81K	2.64 ± 0.72	1.24	10.0	4.70 × 10^–1^	1.24 × 10^–2^	35.0	6.90 ± 0.95
R135K	6.19 ± 1.9	28.3	0.5	4.57	1.42 × 10^–2^	35.0	7.02 ± 0.90
D162G	1.43 ± 0.47	9.30 × 10^–4^	1437	6.50 × 10^–4^	1.34 × 10^–3^	40.0	6.94 ± 0.96
R191H	55	2.00 × 10^–2^	2381	3.64 × 10^–4^	4.83 × 10^–2^	40.0	8.09 ± 0.99
Y316F	1.57 ± 0.3	6.83	1.0	4.35	6.83 × 10^–3^	37.0	5.74 ± 0.26
P319S	4.26 ± 1.11	3.62 × 10^–1^	100	8.50 × 10^–2^	3.62 × 10^–2^	35.0	7.56 ± 0.31
E322V	2.81 ± 0.68	3.80	5.0	1.35	1.90 × 10^–2^	37.0	3.99 ± 0.26
*GROUP III*							
E33Q	3.90 ± 0.67	1.98	5.0	5.08 × 10^–1^	9.91 × 10^–3^	25.0	11.1 ± 1.24
L121I	7.12 ± 2.09	7.20E-02	390.0	1.01 × 10^–2^	2.80 × 10^–2^	35.0	8.97 ± 1.40
L200F	3.23 ± 0.4	2.43E-02	500	7.52 × 10^–3^	1.21 × 10^–2^	30.0	5.76 ± 0.86
D235V	3.97 ± 0.67	3.10E-01	50	7.81 × 10^–2^	1.55 × 10^–2^	37.0	8.60 ± 0.88

^a^The catalytic activity of the P-MAPK1 wild type and variants was determined at the desired temperature with the substrate peptide AQT0490. Kinetic parameters were determined at 30 °C by using at least at 10 different concentrations of AQT0490. *E*a was determined by Eq. (1) in the temperature range between 10 °C and the temperature of maximal activity (T_max_) for each protein. Data are reported as the mean ± SE of the fit

**Table 6:** Kinetic parameters and effect of temperature on kinase activity of MAPK3 wild type and variants^a^

	*K*_m_ (μM)	*k*cat (s^−1^)	[enzyme] (nM)	*k*cat/*K*_m_ (s^−1^ μM^−1^)	*V*_max_ (μM/sec)	*T*_max_ (°C)	*E*a (kcal/mol)
Wild type	3.17 ± 0.59	2.05	8.0	0.65	1.638 × 10^–2^	37.0	4.28 ± 0.28
*GROUP I and II*							
E98K	2.46 ± 0.43	2.65	10.0	1.08	2.65 × 10^–2^	25.0	5.88 ± 1.12
R152W	11.14 ± 1.8	1.80	15.5	0.16	2.79 × 10^–2^	40.0	5.57 ± 0.28
P336Q	3.52 ± 0.49	0.80	25.0	0.23	2.00 × 10^–2^	37.0	6.26 ± 0.81
E339V	2.91 ± 0.59	1.07	10.0	0.37	1.07 × 10^–2^	30.0	4.81 ± 0.71
*GROUP III*							
I73M	5.30 ± 0.80	1.71	15.0	0.32	2.56 × 10^–2^	40.0	4.83 ± 0.38
Q79H	3.31 ± 0.44	0.16	122	0.05	2.01 × 10^–2^	25.0	8.61 ± 1.82
A160T	3.41 ± 0.72	2.06	8.00	0.60	1.65 × 10^–2^	25.0	9.32 ± 1.74
T198I	3.27 ± 0.45	10.74	40.0	3.28	4.30 × 10^–1^	25.0	8.16 ± 1.87
E214D	8.51 ± 1.20	0.49	90.0	0.06	4.40 × 10^–2^	37.0	8.75 ± 0.83
L281I	2.23 ± 0.37	0.93	10.0	0.42	9.27 × 10^–3^	30.0	10.2 ± 0.59
V290A	14.49 ± 1.60	1.27	50.0	0.09	6.34 × 10^–2^	20.0	12.7 ± 1.95
R359W	3.17 ± 0.48	1.47	10.0	0.46	1.47 × 10^–2^	35.0	6.37 ± 0.68
E362K	6.55 ± 0.52	1.14	20.0	0.17	2.28 × 10^–2^	25.0	11.1 ± 0.52

^a^The catalytic activity of the P-MAPK3 wild type and variants was determined at the desired temperature with the substrate peptide AQT0490. Kinetic parameters were determined at 30 °C by using at least at 10 different concentrations of AQT0490. *E*a was determined by Eq. (1) in the temperature range between 10 °C and the temperature of maximal activity (T_max_) for each protein. Data are reported as the mean ± SE of the fit

The comparison of the temperature for maximal activity (Tmax) and of the activation energy (*E*_a_) of the MAPK1 and MAPK3 wild type shows that the activation energy for this latter is about one half than that of MAPK1, with a Tmax 7 °C higher (Tables 5, 6). The *E*_a_ of MAPK1 group I, II and III is very similar to the wild type except for Y316F, E322V and L200F reduced to about one half the value of the wild type (Table 5). As far as concern temperature for maximal activity of MAPK1 variants, only L200F shows the same Tmax of the wild type and only E33Q shows a Tmax value 5 °C lower; all the other display a Tmax at least 5 °C higher with D162G and R191H being 10 °C higher than the Tmax of the wild type (Table 5). In the case of MAPK3, the activation energy of I73M, R359W in group III, and those of all the variants in group I and II are very similar to the wild type, whereas all the other activation energies of the variants are larger than that of wild type, ranging from the double to three-times (Table 6). Notably, all the MAPK3 variants show a Tmax for activity lower than that of the wild type with the only exception of I73M, R152W, E214D and P336Q whose Tmax was similar or slightly higher.

### Molecular dynamics

Data analysis was performed on the entire set of wild-type proteins and variants (both phosphorylated and non-phosphorylated) using the GROMACS built-in functions and home-made codes. The error on each of the average values is the standard deviation. A more in-depth analysis was conducted for the variants subjected to experiments, specifically P- and NP-MAPK1 (E33Q, E81K, R135K, D162G and E322V) and P- and NP-MAPK3 E98K.

In Additional file 1: Fig. S6 A, we present the average root-mean-square displacement (RMSD) values, along with their relative errors, calculated from the final 100 ns of the MD simulations. During this period, the RMSD for all samples reached a plateau; hence, also all the subsequent analysis is performed on the last 100 ns of the MD simulations. Interestingly, both active and inactive forms of MAPK1 and MAPK3 wild-type proteins exhibit smaller RMSD values compared to all the variants. This observation implies that the variants’ structures differ more significantly from the initial crystal structure, regardless of the specific point mutation that was introduced.

In Additional file 1: Fig. S6 B, we show the plot of the gyration radius (Rg) and its corresponding error for all the simulated structures. The Rg of P-MAPK1 wild-type protein is smaller than that of NP-MAPK1 one, meaning that the active MAPK1 wild-type protein is more closed than the inactive one. On the other hand, the Rg of P-MAPK3 wild type is larger than both NP-MAPK3 and P-MAPK1 wild-type proteins (the active MAPK3 is more opened than the other structures). Regarding the variants, NP-E322V exhibits a smaller Rg compared to the inactive wild-type protein and is comparable to that of the active one. Both P-E81K and P-E322V have a larger Rg than P-MAPK1 and are comparable to NP-MAPK1 wild type. However, for the variant P-E98K of MAPK3, it shows a smaller Rg in comparison with P-MAPK3 wild type and its homologous variant (P-E81K). These findings highlight the structural differences among the variants and provide valuable insights into their dynamic behaviors during the simulations.

**Fig. 6:**
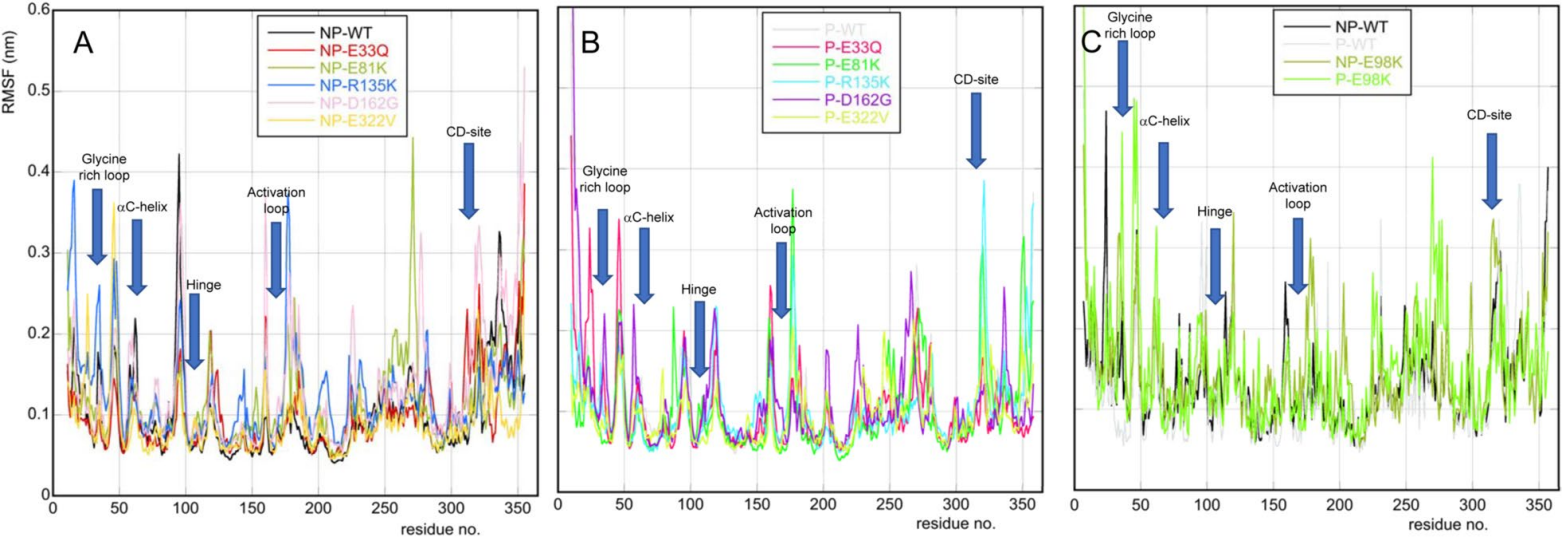
Root-mean-square fluctuations of Ca atoms as a function of the residue number, averaged over the last 100 ns of MD simulation: NP-MAPK1 (panel A), P-MAPK1 (panel B) and NP- and P-MAPK3 (panel C). The arrows indicate interesting proteins’ regions

The root-mean-square fluctuations (RMSF) of the whole set of wild-type and variants proteins for MAPK1 and MAPK3 are shown in Fig. 6 (NP-MAPK1 in panel A, P-MAPK1 in panel B and NP- and P-MAPK3 in panel C), with interesting regions highlighted. Comparing MAPK1 and MAPK3 wild-type proteins, both active and inactive, we find out that the glycine-rich loop and the αC-helix are slightly more mobile in NP- and P-MAPK1 than in NP- and P-MAPK3 wild-type proteins, while the CD-site is the most mobile in P- and NP-MAPK3 wild-type ones. NP- and P-E98K variants are both more mobiles than NP- and P-MAPK3 wild type in the glycine-rich loop, and P-E98K is the least mobile of MAPK3 proteins in the CD-site region. Moreover, comparing the different panels of Fig. 6 it appears that NP- and P-E98K variants are the proteins which experience the most significant fluctuations in their atomic positions. The increased mobility of E98K variants might have functional implications and could be related to its specific biological role and interactions with other molecules or substrates. Regarding NP-MAPK1 variants compared with the inactive wild type, the glycine-rich loop of NP-R135K is the most mobile, the αC-helix region is more mobile in the wild-type protein than in the variants, while the 185–187 region is more mobile in the variants than in the inactive wild type. Lastly, the CD-site is the most mobile in NP-D162G variant. The glycine-rich loop of P-MAPK1 wild-type protein is more mobile than in the variants, the DFG sequence is more mobile in P-D162G variant than in the other P-MAPK1 proteins, and P-E81K and P-R135K are the two most mobile P-MAPK1 variants in the CD-site region.

Indeed, MD simulations provide valuable atomistic information on proteins, enabling us to investigate important structural characteristics. In the present study, we calculated the time evolution along the MD simulation of the distance between the residues K54/K71 and E71/E88 in MAPK1/MAPK3, which form a salt bridge in the active proteins, and the solvent-accessible surface area (SASA) for the activation lip (185–187 in MAPK1 and 202–204 in MAPK3) residues, the CD-site region and the F-site residues.

As expected, the K54/K71-E71/E88 distance is smaller in the phosphorylated (active) wild-type proteins (Additional file 1: Fig. S7) than in the non-phosphorylated (inactive) ones. The P-MAPK1 variants, all show a larger distance than the one of the active wild type between the two residues (comparable with that of the NP-variants), except for those variants in which the CD-site was modified, namely P-D321G, P-D322N, P-E322K and P-E322V (the latter is the only one shown in the present work). All the NP-MAPK1 variants exhibit a distance comparable to or larger than that of NP-MAPK1 wild type, and similar trends are observed for NP-MAPK3 and its variant. Notably, the K71-E88 distance in the P-E98K variant shows a similar magnitude to that of P-MAPK3 and P-MAPK1 wild-type proteins, indicating that the E98K mutation might have a limited effect on the present salt bridge, at variance with what happens for P-E81K, that shows a larger distance than the P-MAPK1 wild type.

The SASA of the activation lip residues (Additional file 1: Fig. S8 A) was found to be larger in phosphorylated than in non-phosphorylated proteins. Notably, there were no significant differences in its value among all the P-MAPK1 samples. However, the P-E98K variant of P-MAPK3 exhibited a slightly smaller SASA compared to both P-MAPK3 wild-type protein and all the P-MAPK1 proteins. Moving on to the non-phosphorylated variants, the SASA values of the activation lip in NP-variants are all slightly larger than that of the NP-MAPK1 wild type except the one of NP-E33Q and NP-E81K variants. The SASA value of the CD-site region (Additional file 1: Fig. S8 B) for both the NP- and P-MAPK1/MAPK3 proteins is comparable; the only variants which exhibit a SASA significantly smaller than the one of their corresponding wild-type proteins are P-E33Q, NP-E81K and NP-E322V. The SASA of F-site residue (Additional file 1: Fig. S8 C) for P-MAPK1 wild-type protein is substantially smaller than both NP-MAPK1 and NP- and P-MAPK3 ones. The P-E98K variant of MAPK3 has a SASA value within the errors of P-MAPK1 wild type, while all the P-MAPK1 variants have a larger SASA than P-MAPK1 wild-type protein except for P-R135K. All the NP-MAPK1 variants, except NP-E322V (whose SASA is within the errors of P-MAPK1 wild type), have a SASA value compatible with the one of NP-MAPK1 wild-type protein.

Finally, we clustered together the proteins’ configurations whose RMSD values differ less than a given threshold (a reasonable choice is 2.5 Å) with the aim of identifying leading structural features. The whole set of proteins has only one representative structure in the last 100 ns of MD simulation (meaning they are stables). In Additional file 1: Figs. S9 and S10, we show a blow-up of the representative structure of each wild-type and variants proteins’ cluster highlighting some interesting amino acids and regions. Together with a picture of the salt bridge (whose values are shown in Additional file 1: Fig.S7), we may observe the D167/D184 direction of MAPK1/MAPK3 proteins which points out from the active site in the inactive wild-type protein and points in for the active one (NP- and P-MAPK1 wild-type proteins are shown Additional file 1: Fig. S9 A; NP- and P-MAPK3 proteins are shown in Additional file 1: Fig. S9 C). The direction of D167 is consistent with that of P-MAPK1 wild-type protein only in the P-R135K variant (shown in Additional file 1: Fig. S10 B), and in the variants not included in the experiments performed here. Among the NP-MAPK1 variants, only the NP-E81K variant (Additional file 1: Fig. S9 B) exhibits the same direction as the active protein (P-MAPK1), along with two variants not considered in this study (NP-D321G and NP-D321N). Both P- and NP-MAPK3 variants maintain the same direction of D184 as their respective wild-type proteins (Additional file 1: Fig. S9 D). The glycine-rich loop points toward the αC-helix in all P-MAPK1 proteins except for P-D162G, while it is the glycine-rich loop of the inactive proteins which points toward the αC-helix for MAPK3 proteins. T185 points toward the αC-helix in all P-MAPK1 proteins, while it is Y205 which points toward αC-helix in P-MAPK3 ones.

## Discussion

The regulation of cellular functions is based on a balanced state between protein kinases and phosphatases. Normal cellular processes are triggered by phosphorylation.

In conditions such as cancer, abnormal phosphorylation plays a significant role in modifying protein structure, function and regulatory mechanisms [40, 45].

MAPK1/3 represents the last step in the signaling cascade of Ras kinase. They are encoded by two different genes on two different chromosomes, and both are quite stable in vivo and usually co-expressed [1]. The serine/threonine kinase MAPK1 and MAPK3 are activated downstream by the Ras-Raf-MEK-ERK signal transduction cascade. Despite the large sequence identity and the fact that they share many, mostly all, their functions, they do not seem functionally redundant; thus, we decided to study them jointly. The MAPK1/3 spectral properties in solution are similar but not identical and both, as suggested by near-UV CD and intrinsic fluorescence spectroscopy, present to a different extent some tertiary structure rearrangements upon phosphorylation of the two tyrosine and threonine residues important for catalysis.

Many somatic missense variants of MAPK1 and MAPK3, found in cancer tissues, are reported in COSMIC database [43] each carrying single mutations in different regions of the sequence. The presence of these somatic variants in cancer tissues may find several explanations, ranging from the genomic instability caused by tumor promoted inflammation and alteration in the control of cell division [39]. These mutations become particularly interesting when they are observed on these proteins, usually playing a pivotal role in the control of cellular functions, since they may alter the protein conformation in solution leading to drastic changes in their functions through the alteration of their interaction with other substrate proteins and ligands [10, 42] and become associated to ERK-cascade inhibitor resistance.

In the selection of the mutants, we tried to explore the whole primary sequence of the two kinases, with the aim to select, when possible, those variants placed in similar points of the two proteins closely identical architecture (Figs. 1 and 2). In addition, we looked for those somatic variants that, at the time of the selection, were frequently observed in cancer tissues and/or were expected to alter the physical and chemical properties of the variants because of the changes in the amino acid residue. To define the position of the mutated residues in each variant of the two proteins we divided them into three groups, depending on their indirect interaction (group I) within the common docking site (CD-site) or on their direct participation in it (group II) or their position in different protein locations. The CD-site, selected for the identification of the three variants groups, is an important energetic spot in the two proteins and the MAPK1 variants directly involved in its function have been extensively studied [24, 44]. Table 7 reports the main differences and/or similarities between the corresponding spectral properties, pathways for unfolding, thermal stability and activity temperature dependence and catalytic efficiency for the MAPK1/3 wild type and the active and non-active forms of the single nucleotide variants selected for this study.

**Table 7:** Effect of single amino acid substitution on MAPK1 and MAPK3 variants stability and catalysis

	^a^Tertiary structure changes		^b^Three-state unfolding		^c^Two-state unfolding		^d^ΔTm (°C)		^e^*k*cat/*K*_m_ ratio	^d^ΔTmax (°C)	^d^Δ*E*a (kcal /mol)
	NP	P	NP	P	NP	P	NP	P			
*MAPK1*											
Wild type	–	–	No	No	Yes	Yes	–	–	–	–	–
*Group I and II*											
E81K	Yes	Yes	Yes	Yes	No	No	0	− 0.9	0.39	5	− 3.05
R135K	No	No	Yes	No	No	Yes	0.1	0.1	3.75	5	− 2.89
D162G	Yes	Yes	No	Yes	Yes	No	− 0.5	5.0	5.33 × 10^–4^	10	− 3.01
R191H	Yes	Yes	Yes	No	No	Yes	3.0	3.1	2.98 × 10^–4^	10	− 1.86
Y316F	Yes	Yes	No	No	Yes	Yes	0.1	0.1	3.57	7	− 4.21
P319S	Yes	Yes	Yes	Yes	No	No	− 4.0	0.1	6.97 × 10^–2^	5	− 2.39
E322V	Yes	Yes	Yes	Yes	No	No	2.1	1.0	1.11	7	− 5.96
*Group III*											
E33Q	Yes	Yes	No	Yes	Yes	No	− 0.9	4.0	4.16 × 10^–1^	− 5	1.15
L121I	Yes	Yes	No	No	Yes	Yes	0	7.0	8.28 × 10^–3^	5	− 0.98
L200F	Yes	Yes	No	No	Yes	Yes	− 1.0	1.1	6.16 × 10^–3^	0	− 4.16
D235V	Yes	Yes	No	No	Yes	Yes	− 2.9	6.1	6.40 × 10^–2^	7	− 1.31
*MAPK3*											
Wild type	–	–	No	No	Yes	Yes	–	–	–	–	–
*Group I and II*											
E98K	No	No	Yes	Yes	No	No	6.0	0	1.66	− 12	1.60
R152W	Yes	Yes	Yes	Yes	No	No	3.0	2.9	2.46 × 10^–1^	3	1.29
P336Q	Yes	Yes	Yes	Yes	No	No	− 2.0	− 0.1	3.5 × 10^–1^	0	1.98
E339V	No	No	Yes	Yes	No	No	0.1	1.0	5.69 × 10^–1^	− 7	0.53
*Group III*											
I73M	Yes	Yes	Yes	Yes	No	No	− 1.0	4.9	4.90 × 10^–1^	3	0.55
Q79H	Yes	Yes	Yes	Yes	No	No	5.5	0	7.69 × 10^–2^	− 12	4.33
A160T	Yes	Yes	Yes	Yes	No	No	− 3.9	− 3.0	9.23 × 10^–1^	− 12	5.04
T198I	Yes	Yes	Yes	Yes	No	No	20.5	0.9	5.046	− 12	3.88
E214D	Yes	Yes	Yes	Yes	No	No	14.3	8.9	9.23 × 10^–2^	0	4.47
L281I	Yes	Yes	Yes	Yes	No	No	0.1	0	6.46 × 10^–1^	− 7	5.92
V290A	Yes	Yes	Yes	Yes	No	No	1.0	8.9	1.38 × 10^–1^	− 17	8.42
R359W	Yes	Yes	Yes	Yes	No	No	− 2.9	9.0	7.08 × 10^–1^	− 2	2.09
E362K	Yes	Yes	Yes	Yes	No	No	− 1.9	− 1.1	2.61 × 10^–1^	− 12	6.82

^a^Observed by comparison of the near-UV CD spectra of the variants with that of the wild type. NP and P indicate the non-phosphorylated and phosphorylate conformation of the proteins, respectively

^b^GdmCl equilibrium unfolding fitted to Eq. (4)

^c^GdmCl equilibrium unfolding fitted to Eq. (6). *T*_m_ is the melting temperature; *T*_max_ is the temperature for maximal activity; *E*_a_ is the activation energy obtained from Eq. (1)

^d^Δcorresponds to the difference between the variant and the wild type

^e^*k*cat/*K*_m_ ratio = *k*cat/*K*_m_variant/*k*cat/*K*_m_wild type

The investigation of the variants spectral properties reveals that an important effect of the single residue mutation in all the variants is an alteration in the protein dependence tertiary arrangements which in turn is responsible for all the other observed changes spanning from the stability changes to the variation in functional activities (Table 7). In the case of MAPK1 D162G and D235V, the spectral changes are notable and both mutations affect two aspartates. D162 is placed in a coil region, before the activation loop (Fig. 1) in proximity to the D-recruitment site (D-site) [32], and is H-bonded to N158 in the NP- and in the P-state conformation and to T160 in the active (Additional file 1: Fig. S11). The MAPK1 D-site, corresponding to residue T159, T160, D318, D321, L115, L121, L157, H125 and Y128, is placed at the opposite site of the catalytic cleft and is formed by negatively charged and hydrophobic residues. In the variant D162G, the mutation of a charged residue into a small glycine may significantly alter the tertiary contacts in a region involved in important interactions of the MAPK1 protein with substrate proteins and as also turns out from MD simulations results where we notice the absence of the K54-E71 salt bridge and a different orientation of the glycine-rich loop. Notably, these changes reflect on the thermal stability of this variant, increased of 5 °C and on its catalytic efficiency, significantly reduced with respect to the wild type, as well as on its Tmax for activity which results 10 °C higher than that of the wild type (Table 7). The D235 residue, placed in a protein region not directly involved in enzyme activity or in substrate recognition, is involved in H-bonds with K 231, N238 and H239 either in the active or in the inactive form (Additional file 1: Fig. S12). In this case the changes in tertiary structure, following the mutation of a charged aspartic residue into a bulky and hydrophobic valine, are not accompanied by a stabilization of an unfolding intermediate with a modification in a more than two-states unfolding pathway, but the thermal stability of the P-state is increased of more than 6 °C, the Tmax for activity of 7 °C and the catalytic efficiency (*k*_cat_/*K*_m_) significantly reduced with respect to the wild type (Table7). In the MAPK3 variants the tertiary structure changes in P336Q are remarkable, as expected upon a P to Q mutation placed in the middle of the CD-site that suppresses an important hydrophobic interaction of P336 with Y334 (Additional file 1: Fig. S13). The unfolding pattern is changed, decreased the thermal stability of the variant, as well as the catalytic efficiency. Notably, in the NP-P319S, the corresponding mutant of MAPK1, the functional changes observed are very similar, as well as the hydrophobic interaction made by the P319 residue with Y317, corresponding to Y334 of MAPK3. The tertiary structure changes observed in MAPK3 E214D are accompanied by notable changes in the enzyme activity and stability parameters: the catalytic efficiency ratio is dropped, the activation energy is doubled, and the thermal stabilities of both the active and inactive states increase of 8.9 and 14.3 °C, respectively. The E214 is involved in a salt bridge with R318 and H-bonded to N218 and S219 (Additional file 1: Fig. S14); these electrostatic interactions in principle should be preserved upon the conservative *E* to *D* mutation, and thus the changes observed may be ascribed to the glutamate substitution with the shorter aspartate that influences these interactions.

The analysis of the *T*_m_ for the two similar kinases shows a significant difference for the two wild type; MAPK1 is about 8 °C more stable than MAPK3 which in terms of temperature for maximal activity is 7 °C higher than MAPK1, as also is shown in the resulting higher mobility of the CD-site on MAPK3 proteins, especially for their E98K variant. The 6 °C increase in *T*_m_ observed for MAPK3 NP-E98K is paralleled by the 3 °C increase observed with R152W and, interestingly, since the arginine 152 makes an electrostatic interaction with E98 (Additional file 1: Fig. S15). The *T*_max_ variations for these two MAPK3 variants are notable: 3 °C increase in *T*_max_ for R152W, whereas for E98K the Tmax is decreased of 12 °C (Table 7). The increase in *T*_m_ is observed for MAPK1 P-E33Q and P-L121I. In the case of E33, this residue, placed in the glycine-rich loop (Fig. 1), is hydrogen bonded to Y30 and makes an electrostatic interaction with R15; then, the substitution with a yet polar and non-charged glutamine may contribute to the thermal stabilization of this variant (Additional file 1: Fig. S16). Notably, the Tmax for E33Q activity is decreased of 5 °C with respect to the wild type, suggesting that the substitution in a region important for the stabilization of ATP binding is not advantageous for the enzyme activity at increasing temperature. The MAPK1 L121 is involved in a hydrophobic interaction with I126; the mutation into isoleucine yields an increase in thermal stability and thermal activity, probably due to a better hydrophobic interaction (Table 5). In MAPK3 group III, a remarkable example is represented by P-V290A and P-R359W because their thermal stability is increased of about 9 °C, but their thermal activity is decreased of 17 and 2 °C, respectively. In the case of P-V290A, the loss of the hydrophobic interaction between V290 and L295 (Additional file 1: Fig. S17), both residues are in the proximity of the F-site, leads to the largest decrease in *T*_max_ observed for all the variants (Table 7). The MAPK3 R359W variant in the active form is significantly more thermostable and slightly less thermally active than the wild type. The R359 is engaged in a salt bridge with E362, and when this latter is mutated into a lysine, the variation observed in thermal activity is larger and leads to a 12 °C decrease with respect to the wild type. Both residues are involved in a complex network of electrostatic and hydrophobic interactions with other residues, as depicted in Additional file 1: Fig. S18.

Among the MAPK1 variants, only the Y316F shows a two-states unfolding transition like that of the wild type with comparable thermodynamic stability parameters. Most probably the change of a tyrosine into a phenylalanine does not alter the network of hydrophobic interactions established by Y316 with Y128 and Y131, as confirmed by the increase in maximal temperature for activity and by the small decrease in activation energy. The analysis of this set of variants suggests that mutation of residues in the CD-site, or interacting with it, perturbs the MAPK1 unfolding thermodynamics, leading to the population of an intermediate, whereas other regions of the protein seem to be more resilient to the effect of the mutations, in terms of equilibrium unfolding. The CD-site modifications turn out to influence more the proteins dynamics, as suggested also in the MD simulations. Interestingly, in the case of MAPK3, for all variants the unfolding transitions are different than the wild type, and the mutations lead to the stabilization of an unfolding intermediate. This evidence strongly indicates that the mutations significantly alter the enzyme thermodynamic states, particularly for MAPK3.

The changes in catalytic efficiency of group I and II MAPK1 and MAPK3 variants are not surprising since these variants are structurally in connection with the CD-site considered as a hot spot in the enzyme structure, involved in interactions with protein substrates and regulators. A significant decrease is observed in the MAPK1 for P-D162G, P-R191H and P-P319S, and in MAPK3 for P-P336Q and P-E339V. The changes in P-D162G, close to the D-site, have been already discussed since the properties of this variants are altered in term of spectral properties, thermal stability and maximum temperature for enzyme activity (Additional file 1: Fig. S11). Indeed, the notable decrease in catalytic efficiency, paralleled by the notable increase of maximal temperature for enzyme activity, observed in P-R191H could be ascribed to the position of the R191, hydrogen and electrostatically bound to the phosphate moiety of Y187 in the catalytic site; hence, the change into a histidine would prevent binding to Y187 leading to a decrease of the enzyme efficiency (Additional file 1: Fig. S19). The P-P319S, P-E322V of MAPK1 and P-P336Q and P-E339V of MAPK3 are all placed at the same relative position in the CD-site (Fig. 1), and the amino acid substitutions may induce the changes in the catalytic parameters through an alteration of the CD-site dynamic. As a matter of fact, P-P319 and P-E322 are reciprocally hydrogen bonded and P319 makes a hydrophobic interaction with Y317 (Additional file 1: Fig. S20). Notably, the temperature for maximal activity of both the MAPK1 variants is increased and the activation energy decreased. In the MAPK3 CD-site the P-P336 and the P-E339 are not directly connected like in MAPK1, but they take part to an extended network of electrostatic interactions (Additional file 1: Fig. S21) with K155, E322 and Y148 and, similarly to MAPK1, there is a hydrophobic interaction between P336 and Y334. In MAPK1 group III there is a notable decrease in enzyme efficiency for P-L200F. This residue is involved in a network of hydrophobic interactions with A260, L237, I255 and L264 (Additional file 1: Fig. S22) that may be critical for the structure of the enzyme during catalysis, as confirmed also by the effect observed on the decrease in activation energy for the enzyme reaction.

## Conclusion

The examination of the changes observed in the somatic variants of MAPK1/3 is characterized by complexity.

The unique common denominator observed in the variations is the tendency to stabilize the native state of the enzyme. Then, the thermodynamic stabilization of the native state, evident in the stabilization of a thermodynamic intermediate upon denaturation, may be expressed in various aspects ranging from an increase in melting temperature or in an increase of the temperature for maximal activity. The alterations in stability and enzyme activity shown by certain MAPK1/3 variants indicate the possibility of a long-range impact on the active site caused by mutations occurring in various regions of the protein, including those far from the catalytic site.

This complexity is not unexpected when studying complex enzymes formed by several domains and based on the formation of an active state upon phosphorylation. The cycle of the enzymes, directed inside the nucleus, and the interactions with proteins substrate and regulator, suggest that the interpretation of the results of the variants in cancer cells in vivo may be complex. The analysis of the impact of this set of mutations on MAPK1 and MAPK3 biophysical and biochemical properties suggests that alternative native state(s) of these proteins may exist and may lead to alternative patterns of interactions with substrates and regulators and/or ligands. Investigating purified MAPK1/3 variants is crucial for comprehending how missense mutations influence both protein function and structure. This research contributes significantly to our understanding of the implications of these mutations. In the context of emerging personalized medicine strategies, it is essential to have a molecular-level understanding of how single nucleotide variations can alter protein structure, function, stability, and interactions.

### Supplementary Information

**Additional file 1**. Supplementary figures and tables.
