# Supplementary material for: The complex impact of cancer-related missense mutations on the stability and on the biophysical and biochemical properties of MAPK1 and MAPK3 somatic variants
Source: Hum Genomics. 2023 Oct 27;17:95. doi: 10.1186/s40246-023-00544-x (PMC10612357; doi:10.1186/s40246-023-00544-x)
Supplement: Supplementary file 1 — Additional file 1. Supplementary figures and tables. [file 40246_2023_544_MOESM1_ESM.pdf]

ADDITIONAL FILE 1

SUPPLEMENTARY INFORMATION

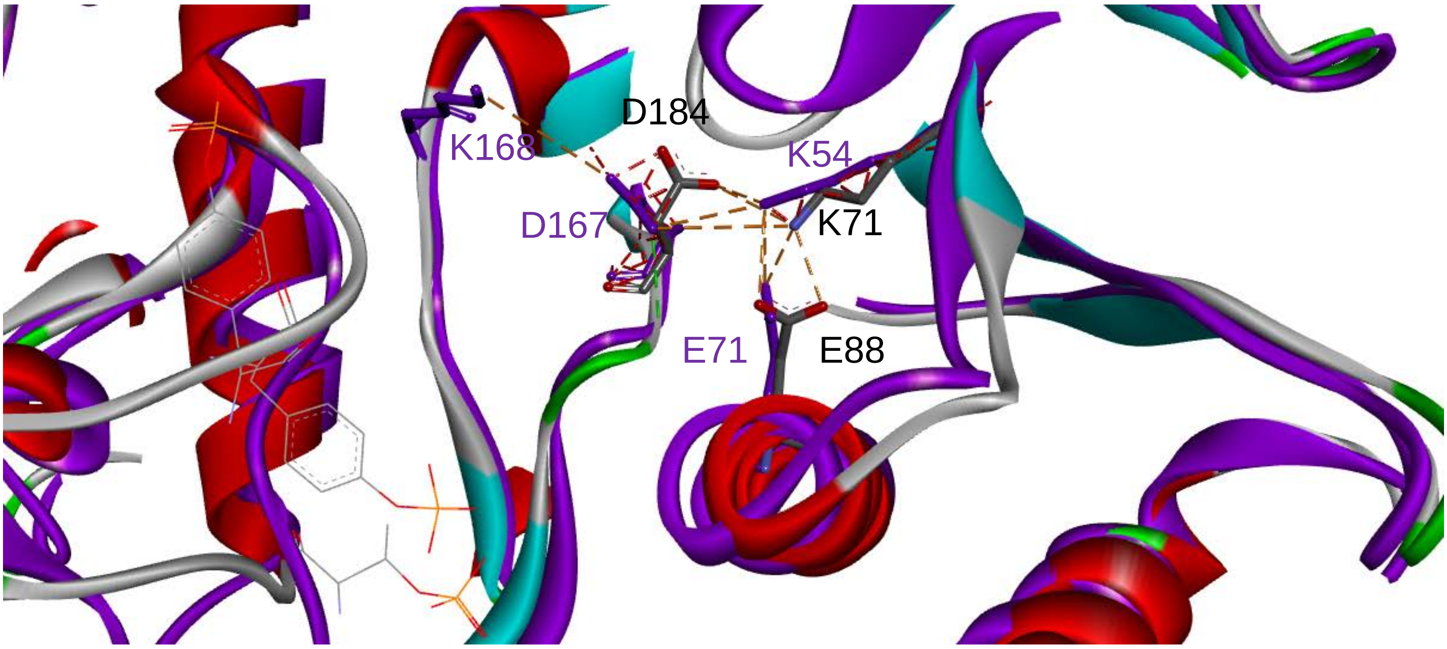

Figure S1. Rearrangement of αC-helix upon phosphorylation of threonine and tyrosine at the active site. Structural comparison of MAPK1 in purple (pdb: 5V60) and MAPK3 (pdb: 2ZOQ), coloured in secondary structure type. The MAPK1 T185/Y187 and MAPK3 T202/Y204, important for the activation of MAPK1/3, are depicted in line. The residues involved in new interactions are in sticks. K168 of MAPK3 catalytic loop is shown in stick.

Table S1. MAPK1 and MAPK3 variants selected from COSMIC

MAPK1	MAPK3
**group I**[a]	**group I**[a]
E81K	E98K
R135K	R152W
D162G	
R191H	**group II**[b]
Y316F	P336Q
	E339V
**group II**[b]	**group III**[c]
P319S	I73M
E322V	Q79H
	A160T
	T198I
**group III**[c]	E214D
E33Q	L281I
L121I	V290A
L200F	R359W
D235V	E362K

[a]group I variants: mutated residue directly interacting with the CD-site; [b]group II variants: mutated residue in the CD-site; [c]group III variants: mutated residue in other regions of the protein.

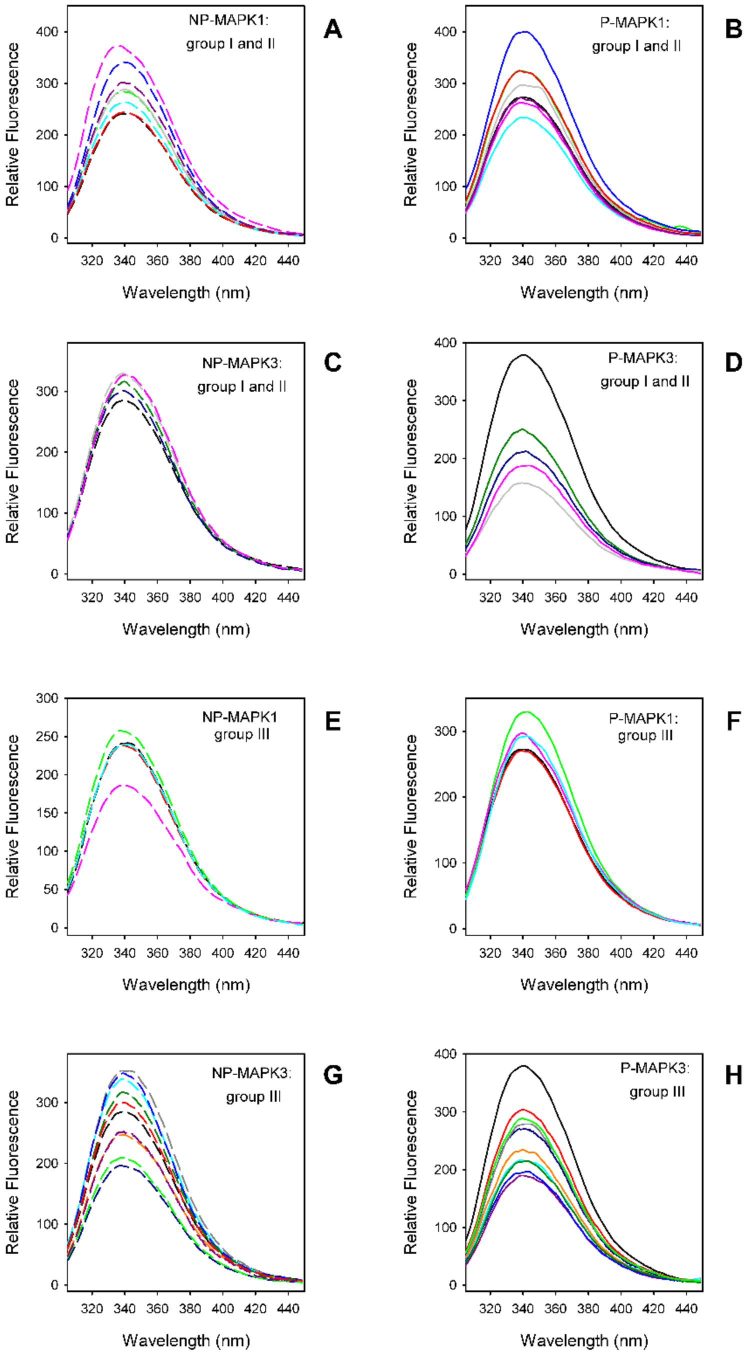

Figure S2. Intrinsic fluorescence emission spectra of nonphosphorylated (NP-) and phosphorylated (P-) MAPK1 and MAPK3 variants.   Fluorescence spectra of group I and II variants of MAPK1 (A, B), MAPK3 (C, D), and group III MAPK1 (E, F) and MAPK3 (G, H) variants were monitored at 130 µg/mL (0.08 AU 280 nm, 295 nm excitation wavelength), in 20 mM Tris-HCl, pH 7.5, 0.1 M NaCl, 0.2 mM DTT. The dashed lines are used for the NP-MAPK1 and N-PMAPK3, the continuous lines are used for P-MAPK1 and P-MAPK3. All spectra were recorded at 20°C.

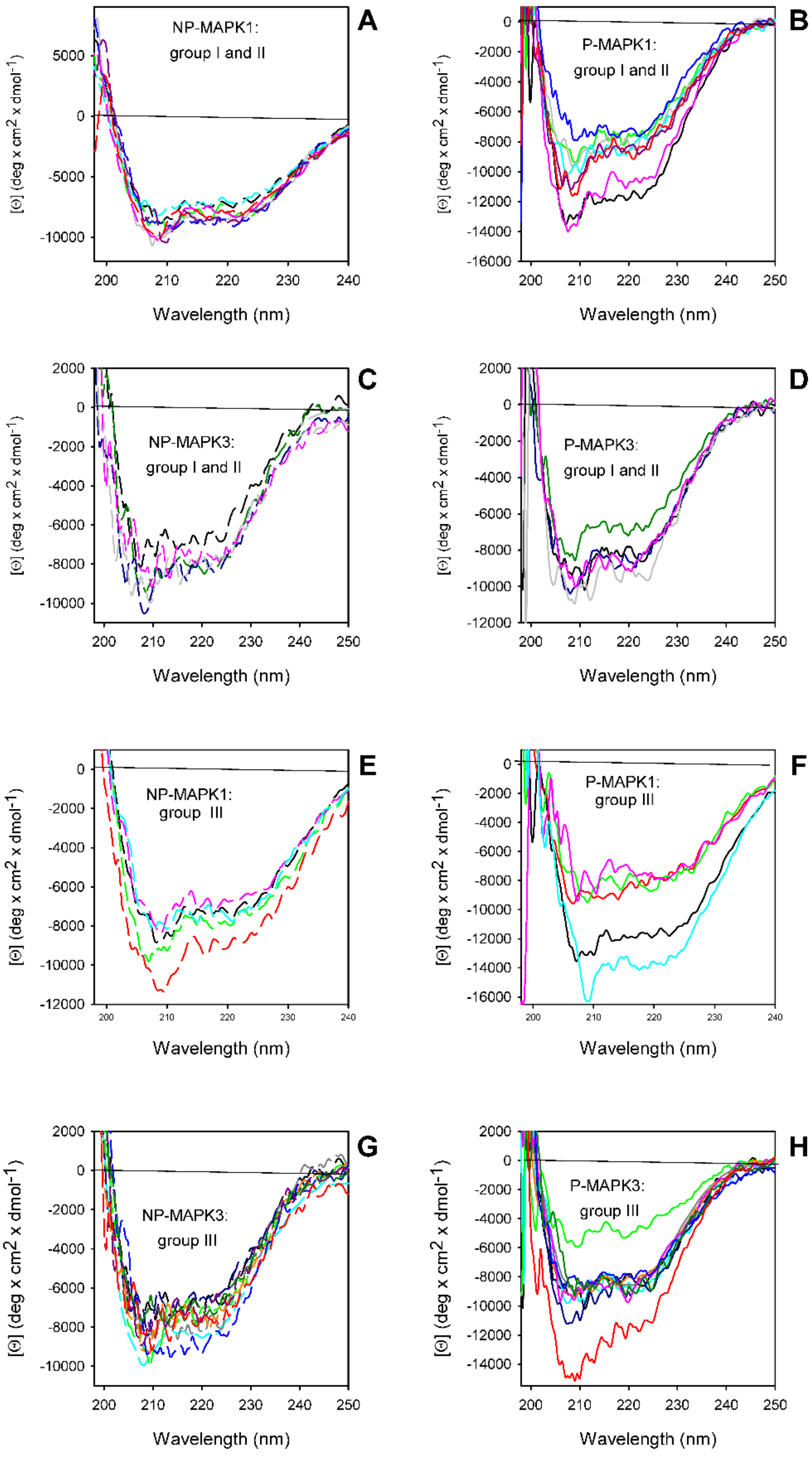

Figure S3. Far-UV CD spectra of nonphosphorylated (NP-) and phosphorylated (P-) MAPK1 and MAPK3 variants. Far-UV CD spectra of group I and II variants of MAPK1 (A, B), MAPK3 (C, D), and group III MAPK1 (E, F) and MAPK3 (G, H) variants were monitored in a 0.1-cm quartz cuvette at 130-170 µg/mL in 20 mM Tris-HCl, pH 7.5, 0.2 M NaCl and 0.2 mM DTT. The dashed lines are used for the NP-MAPK1 and N-PMAPK3, the continuous lines are used for P-MAPK1 and P-MAPK3. All spectra were recorded at 20°C.

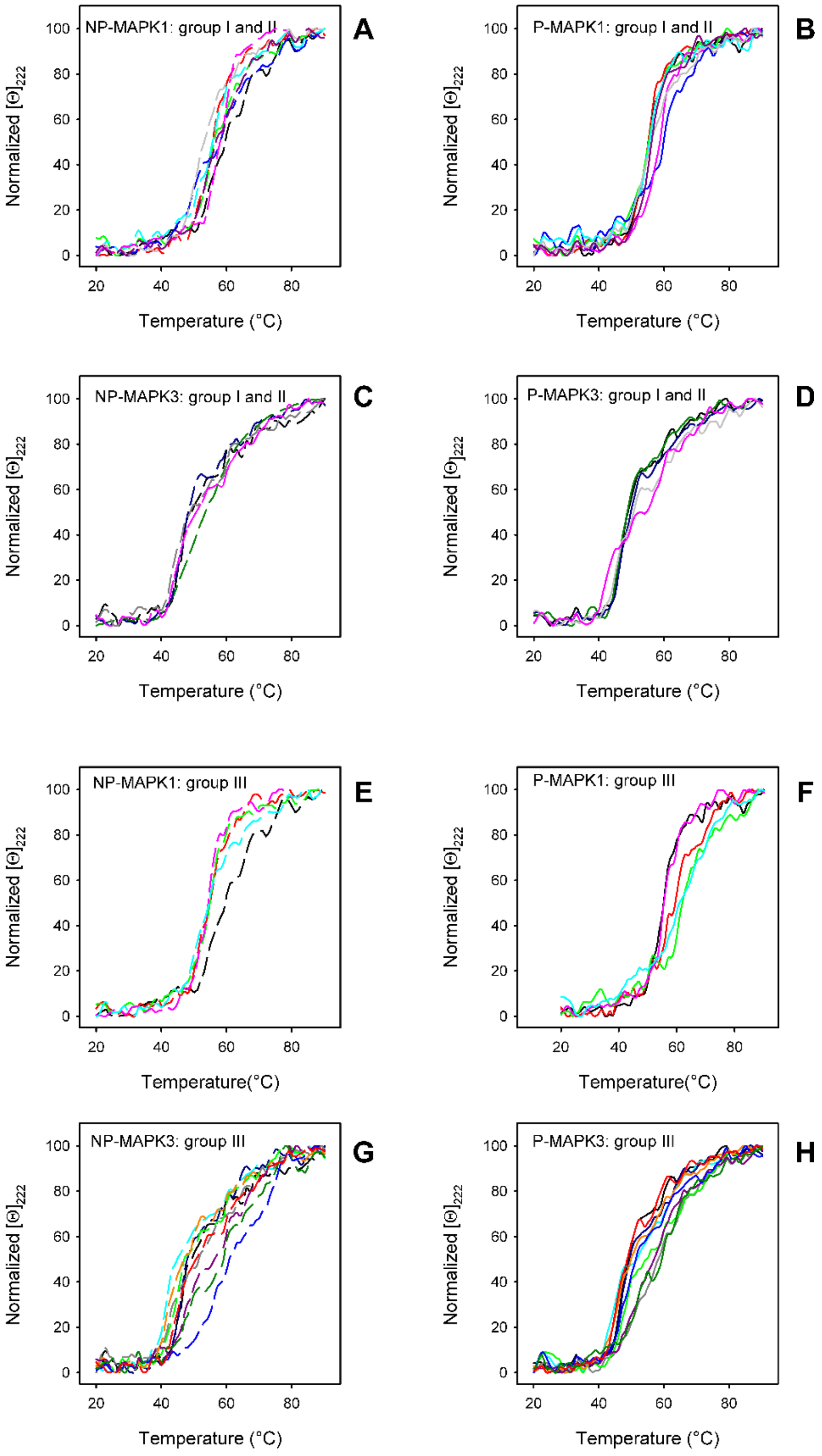

Figure S4. Thermal unfolding of non-phosphorylated (NP-) and phosphorylated (P-) MAPK1 and MAPK3 wild-type and variants. The ERK2 wild-type proteins (100-130 µg/mL), in 20 mM Tris-HCl, pH 7.5, 0.1 M NaCl, 200 µM DTT, were heated from 20°C to 90°C. The molar ellipticity at 222 nm ([Θ]222) was monitored continuously every 0.5 °C and normalized. The first derivative of the thermal transition data is shown in the inset.

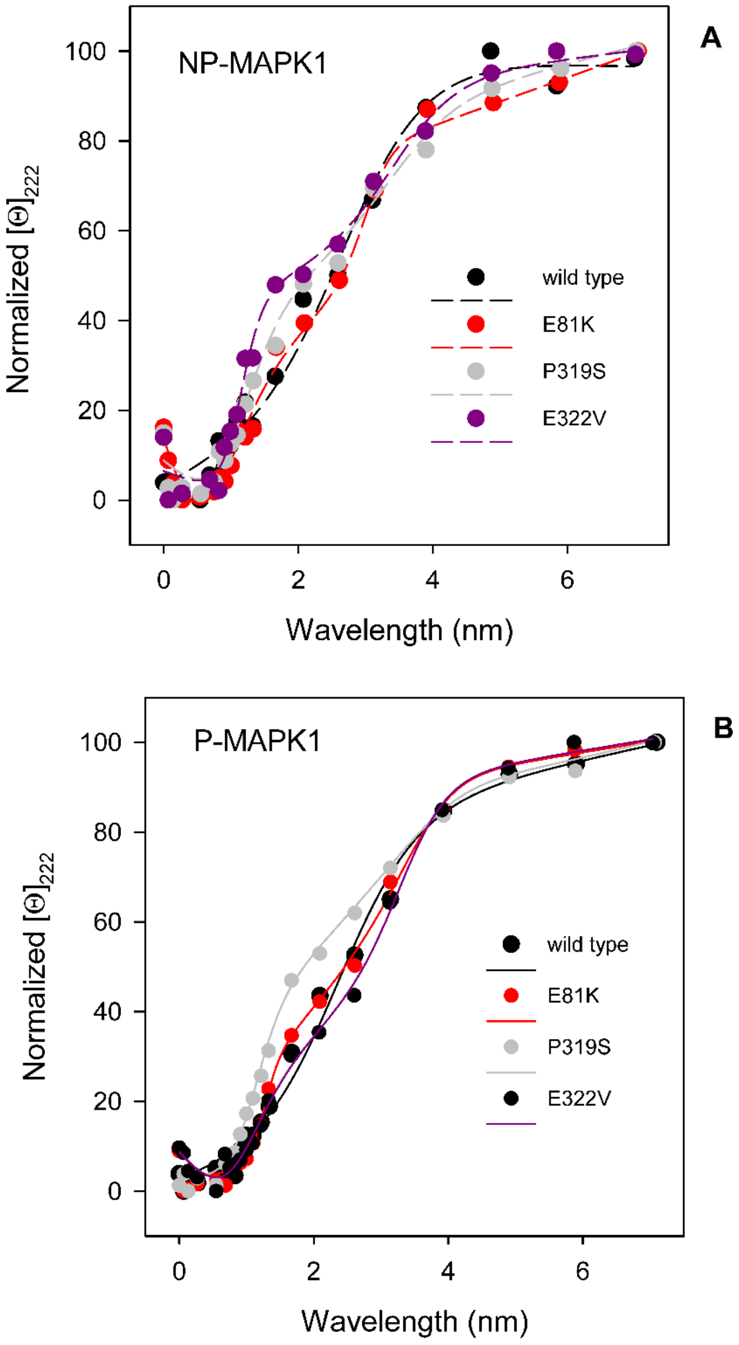

Figure S5. GdmCl-induced equilibrium unfolding of NP- and P-MAPK1 wild type, E81K, P319S and E322V. Normalized molar ellipticity at 222 nm ($[\Theta]_{222}$) of (A)NP-wild type, NP-E81K, NP-P319S and NP-E322V and (B) P-wild type, P-E81K, P-P319S and P-E322V. The dashed (A) and the continuous lines (B) lines represent the nonlinear fitting of the normalized molar ellipticities at 222 nm to Equation (4) (2-state unfolding) for NP-wild type and P-wild type or to Equation (6) (3-state unfolding) for the NP- and P- variants.

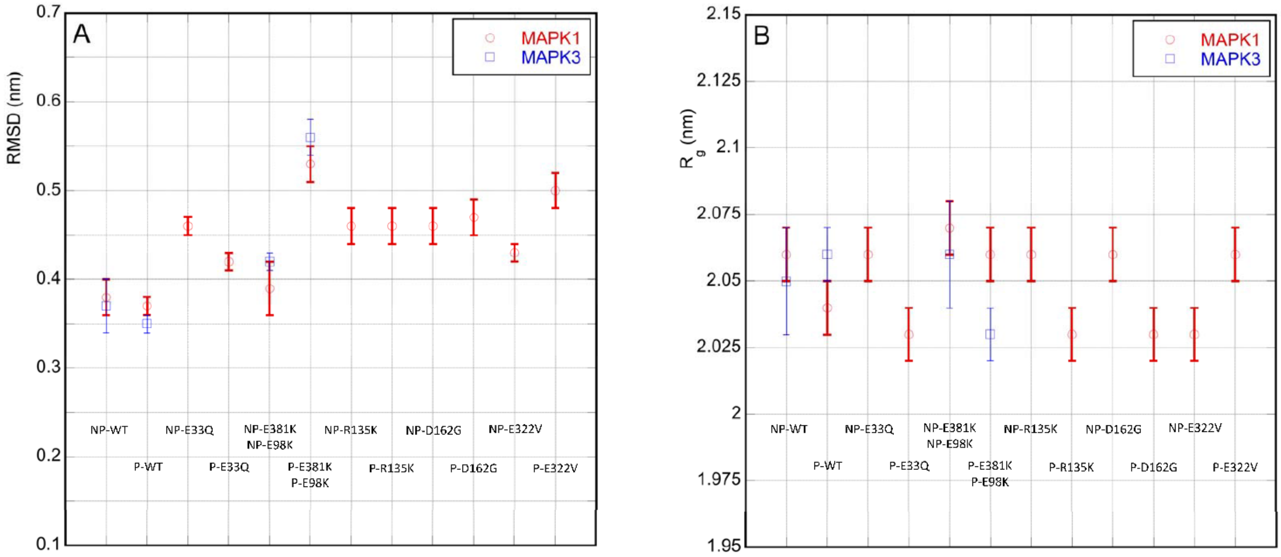

Figure S6. Average values, along with their relative standard deviations, computed over the last 100 ns of MD simulation for root mean square displacement (panel A) and gyration radius (panel B), as a function of the variants' names.

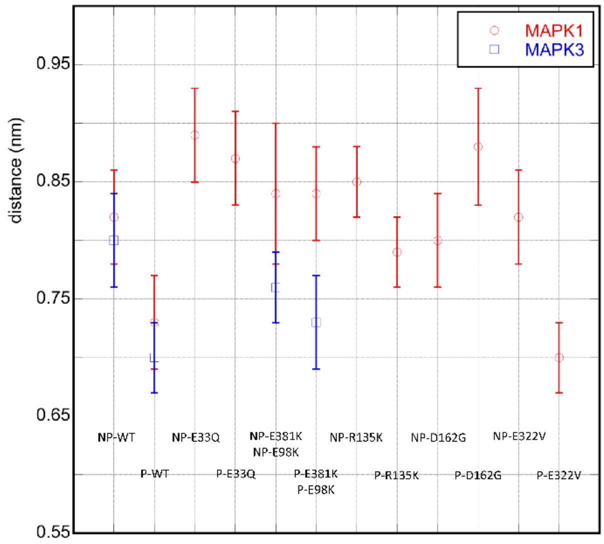

Figure S7. Average values of K54-E71 (K71-E88) distance, along with the relative standard deviations, computed over the last 100 ns of MD simulation as a function of the variants' names.

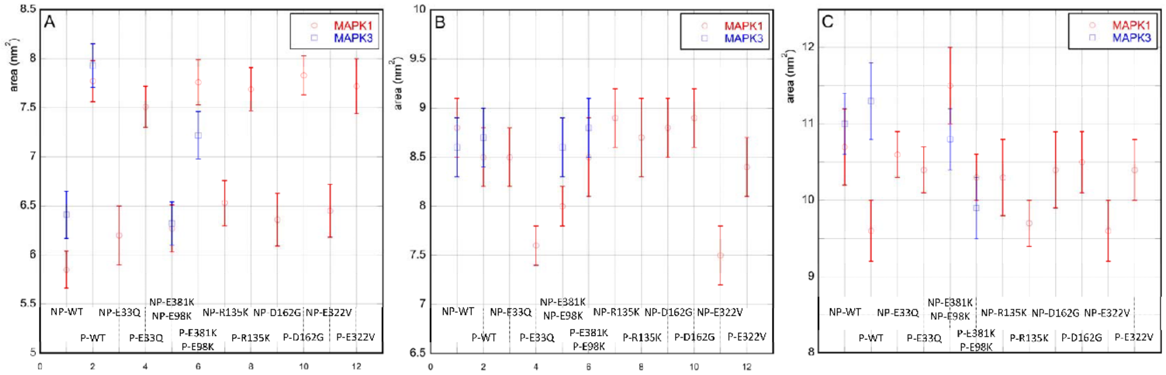

Figure S8. Average solvent accessible surface area values, along with their relative standard deviations, computed over the last 100 ns of MD simulation for the activation lip (panel A), the CD-site (panel B) and the F-site (panel C) residues, as a function of the variants' names.

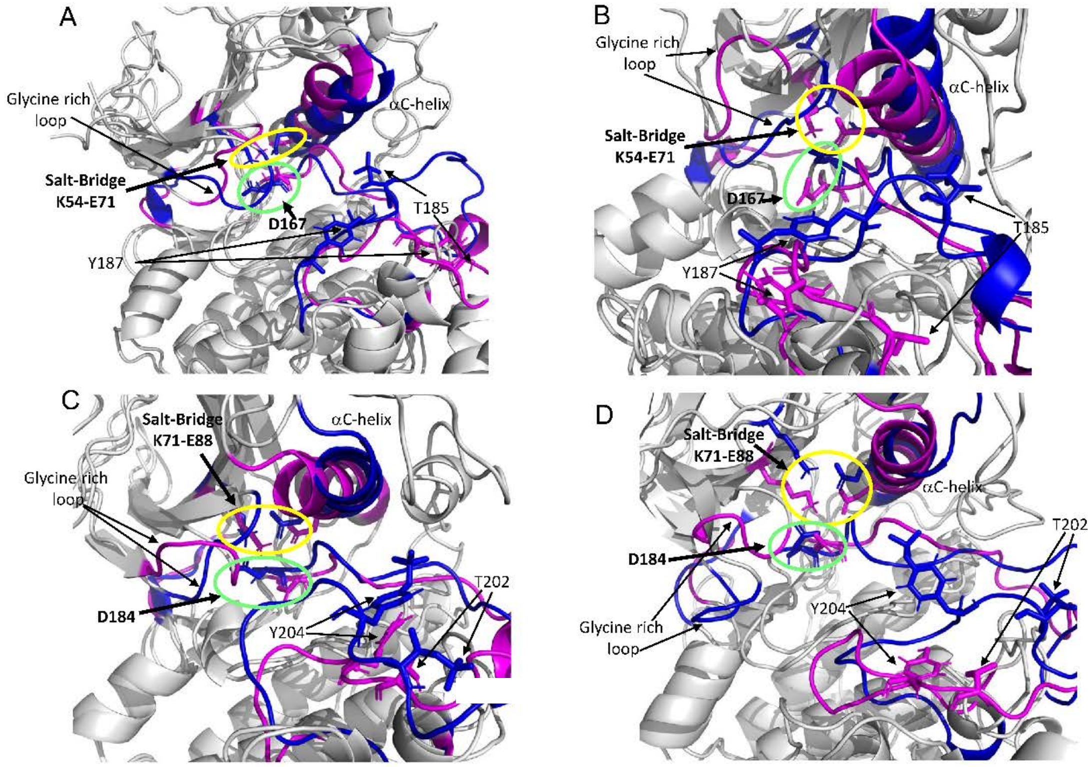

Figure S9. Cartoon representation of the most representative structure of the clusters computed over the last 100 ns of MD simulation (see text). In all the pictures are put in evidence D167 (D184) with a green circle, T185 (T202), Y187 (Y204), the salt-bridge K54-E71 (K71-E88) with a yellow circle, aC-helix and glycine rich loop for P-MAPK1 (P-MAPK3) in blue and for NP-MAPK1 (NP-MAPK3) in magenta. Panel A; P-WT and NP-WT of MAPK1; panel B: P-E81K and NP-E81K of MAPK1; panel C: P-WT and NP-WT of MAPK3; panel D: P-E98K and NP-E98K of MAPK3

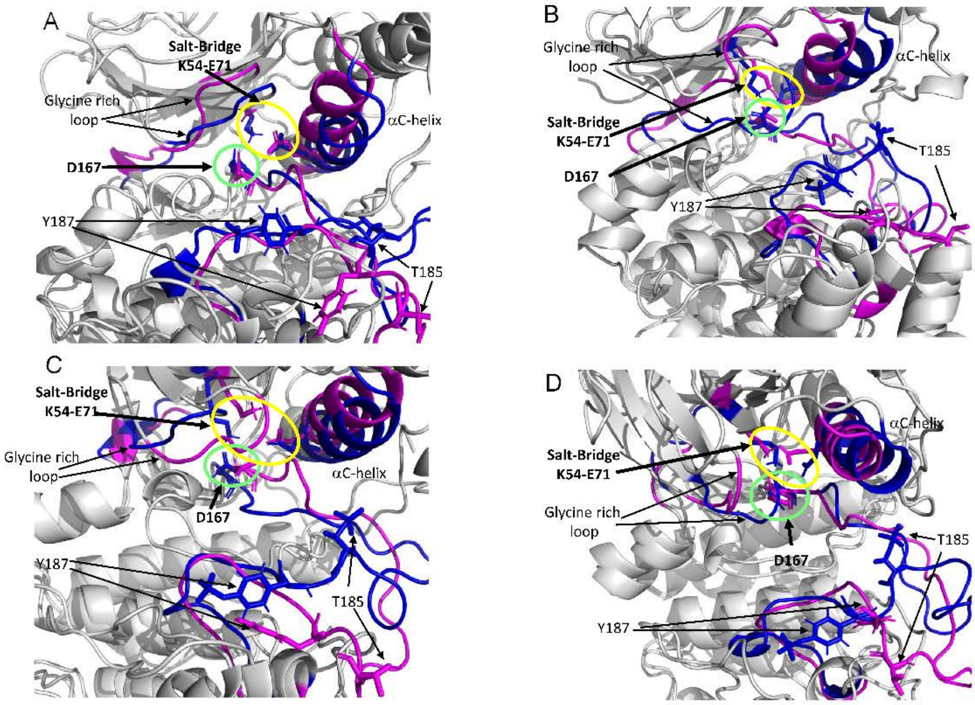

Figure S10. Cartoon representation of the most representative structure of the clusters computed over the last 100 ns of MD simulation (see text). In all the pictures are put in evidence D167 with a green circle, T185, Y187, the salt-bridge K54-E71 with a yellow circle, aC-helix and glycine rich loop for P-MAPK1 variants in blue and for NP-MAPK1 variants in magenta. Panel A; P-33Q and NP-E33Q; panel B: P-R135K and NP-R135K; panel C: P-D162G and NP-D162G; panel D: P-E322V and NP-E322V.

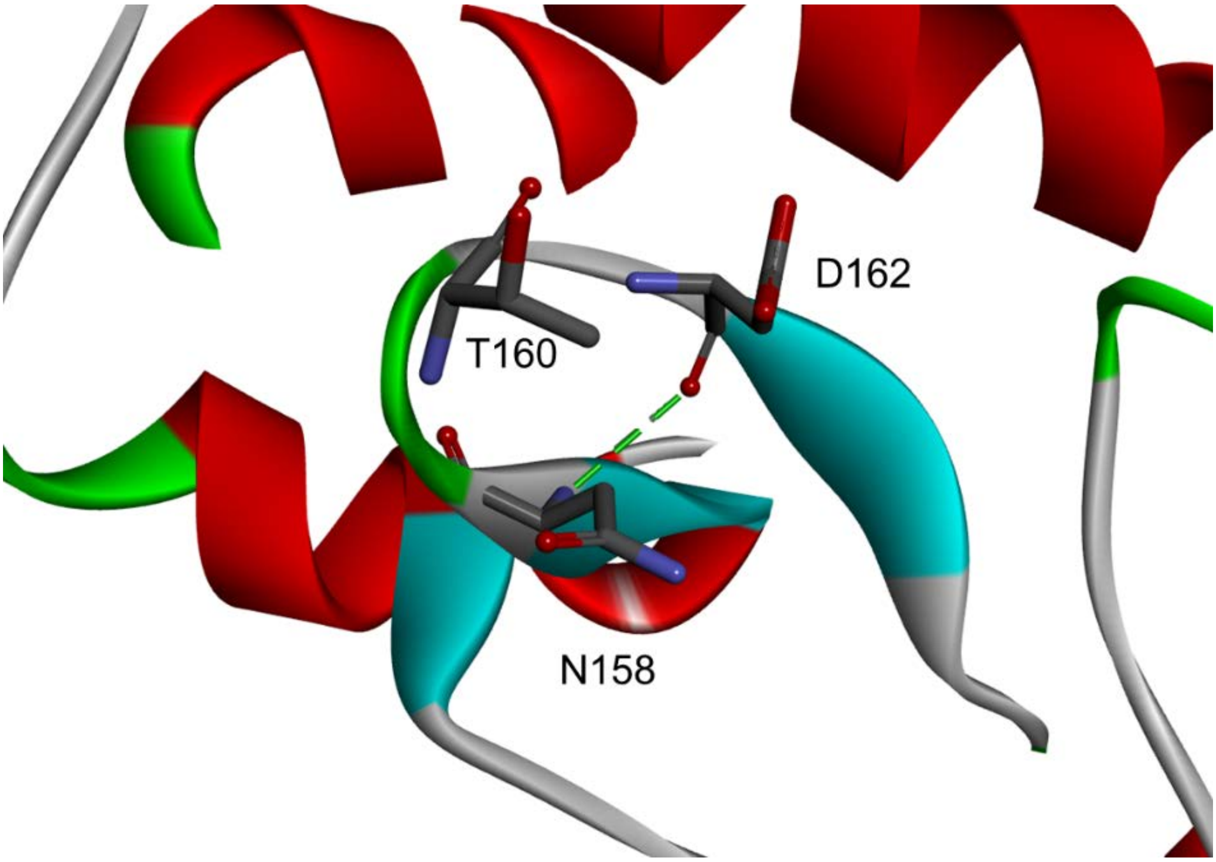

Figure S11. The interaction of D162 residue. MAPK1 inactive form (pdb: 1TVO) is in secondary structure colors. The residues involved in the interactions are depicted in sticks.

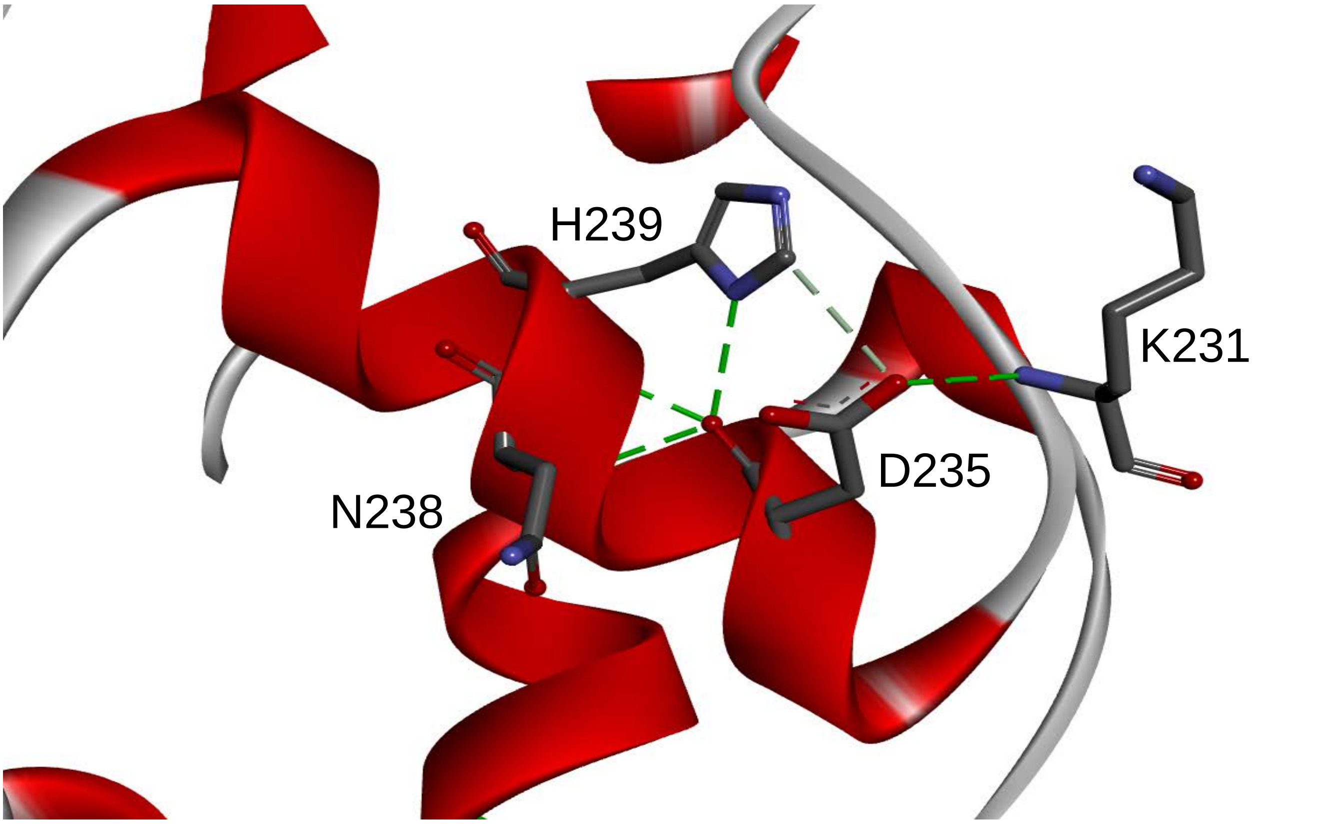

Figure S12. The interaction of D235 residue. MAPK1 inactive form (pdb: 1TVO) is in secondary structure colors. The residues in volved in the interactions are depicted in sticks.

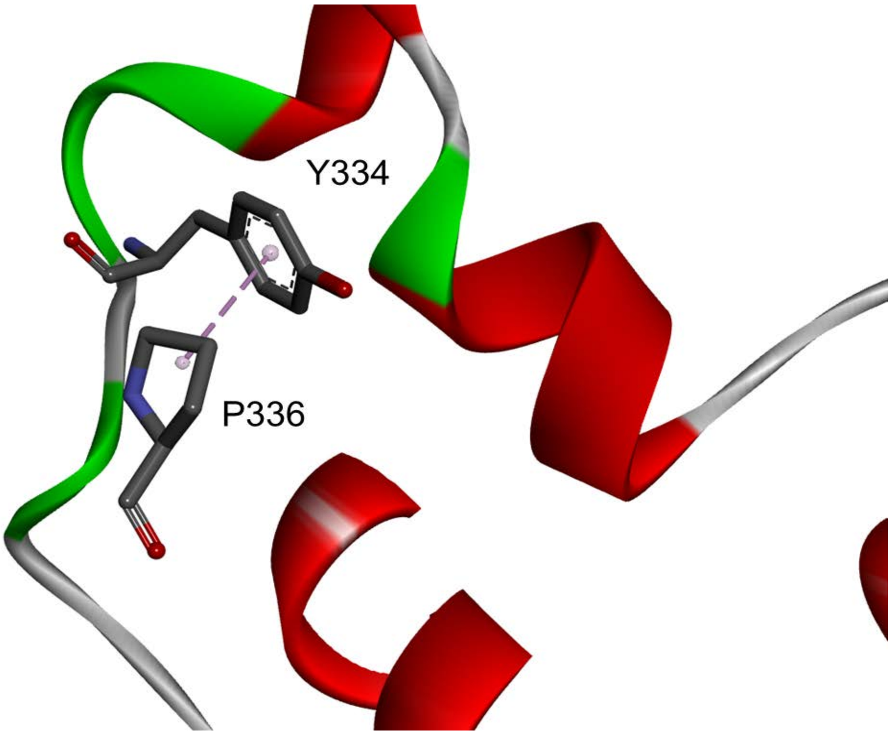

Figure S13. The interaction of P336 residue in the CD-site of MAPK3. MAPK3 active form (pdb: 2ZOQ) is in secondary structure colors. The residues in volved in the interactions are depicted in sticks.

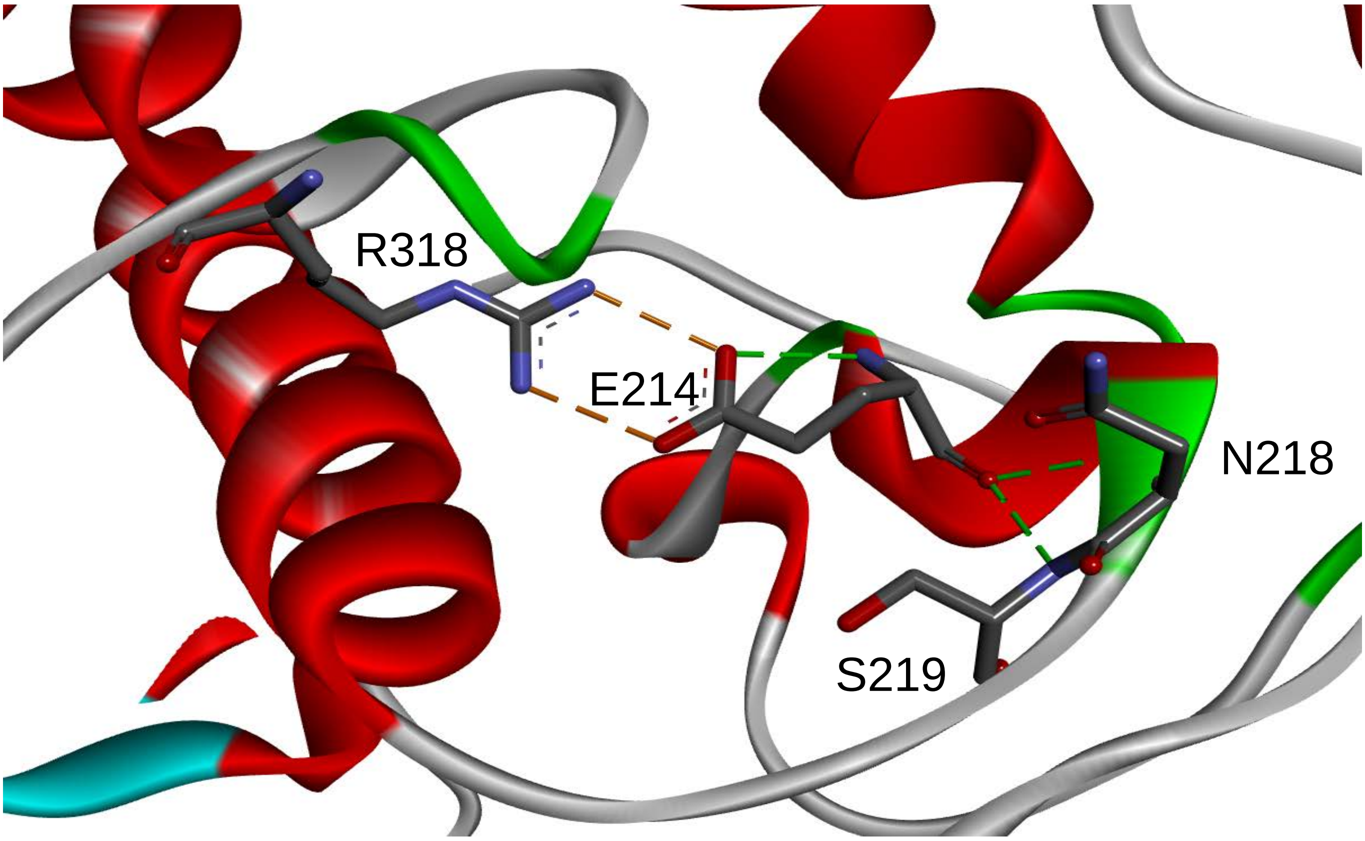

Figure S14. The interaction of E214 residue of MAPK3. MAPK3 active form (pdb: 2ZOQ) is in secondary structure colors. The residues in volved in the interactions are depicted in sticks.

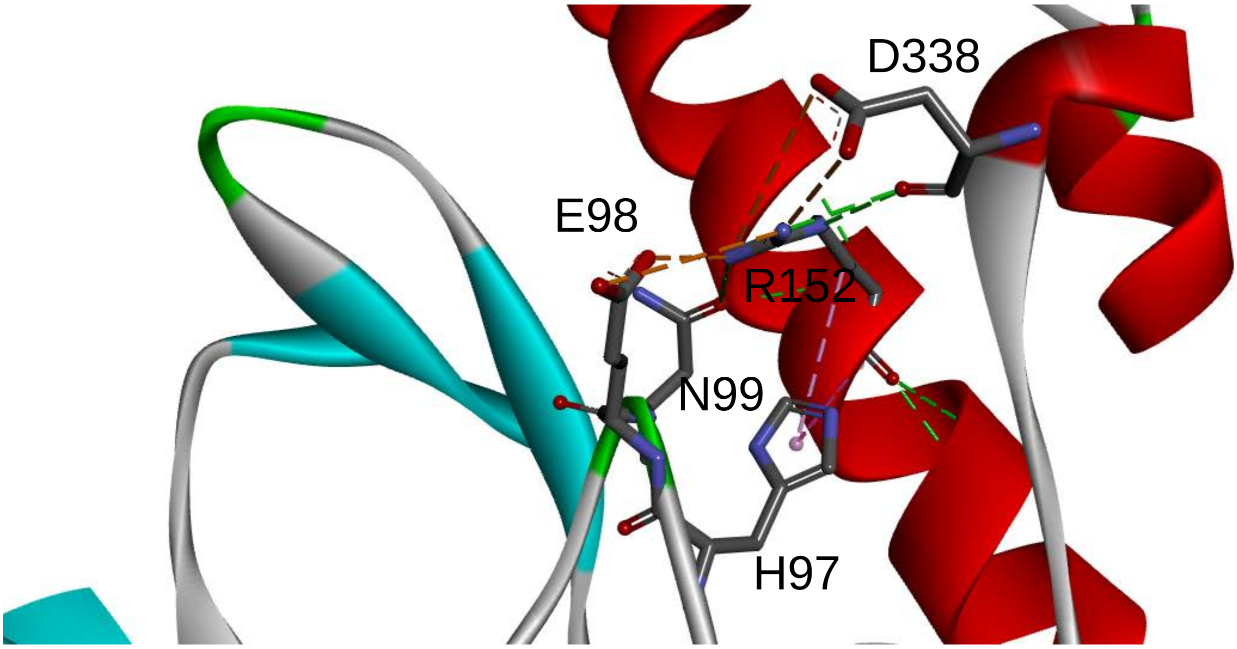

Figure S15. The interaction of E98 and R152 residue of MAPK3. MAPK3 inactive form (pdb: 4QTB) is in secondary structure colors. The residues involved in the interactions are depicted in sticks.

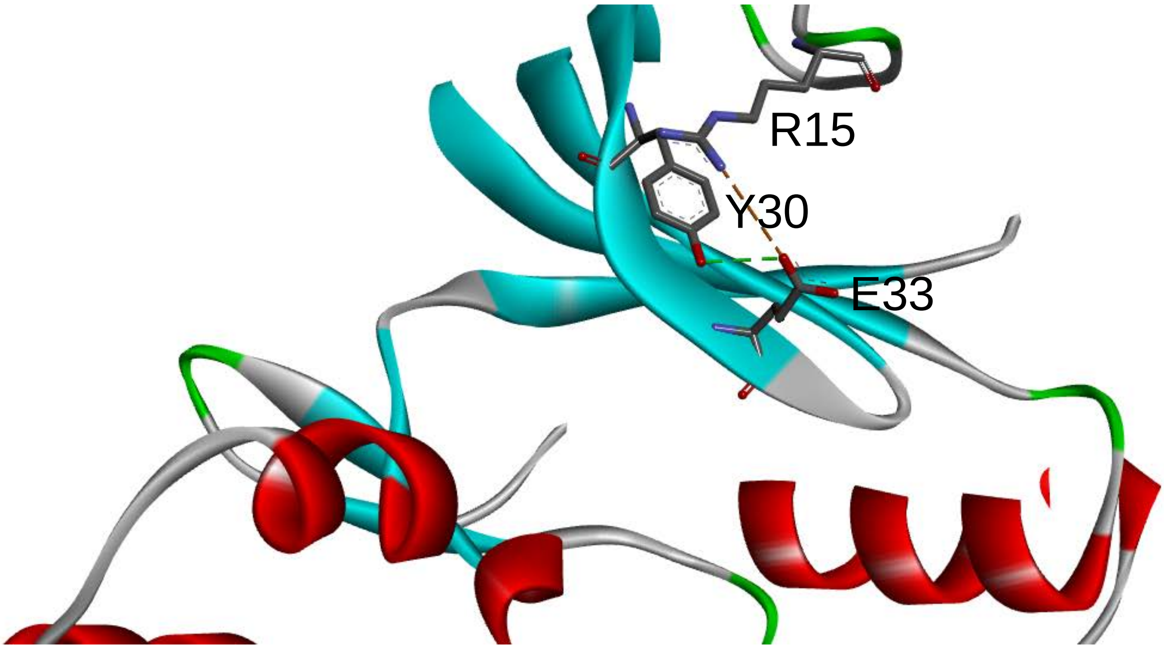

Figure S16. The interaction of E33 and R152 residue of MAPK1. MAPK1 active form (pdb: 5V60) is in secondary structure colors. The residues involved in the interactions are depicted in sticks.

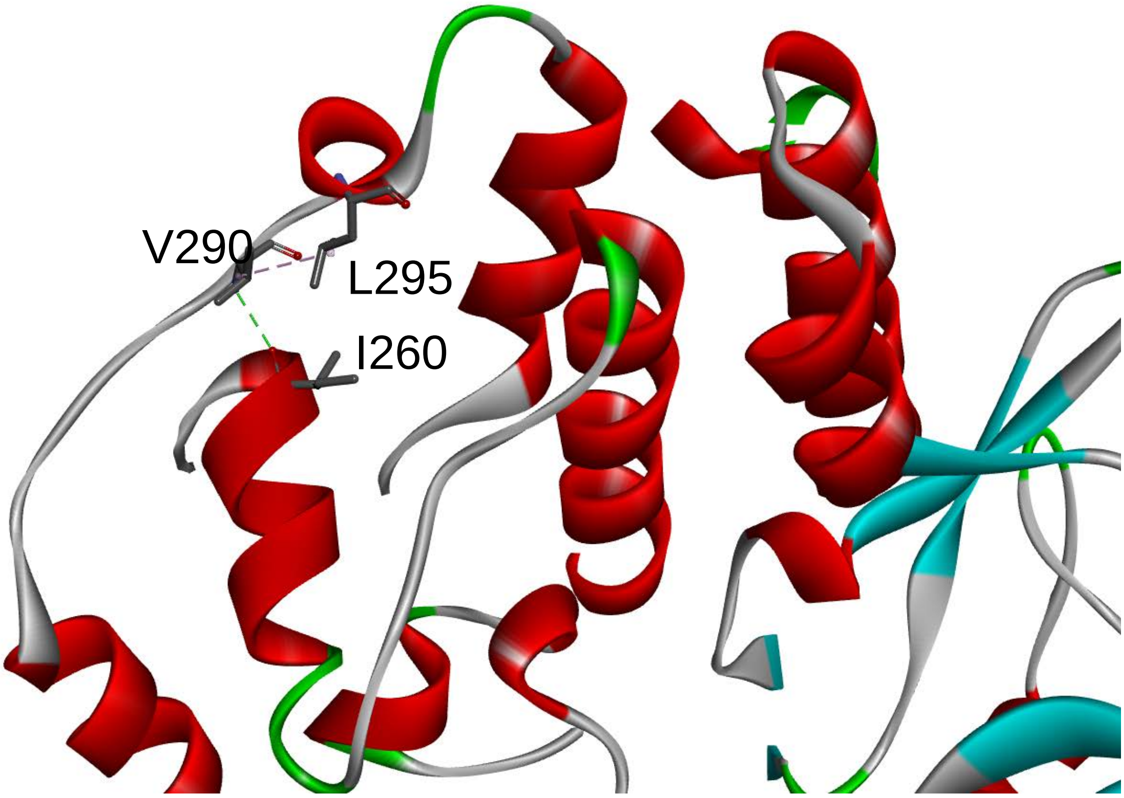

Figure S17. The interaction of V290 of MAPK3. MAPK3 active form (pdb: 2ZOQ) is in secondary structure colors. The residues involved in the interactions are depicted in sticks.

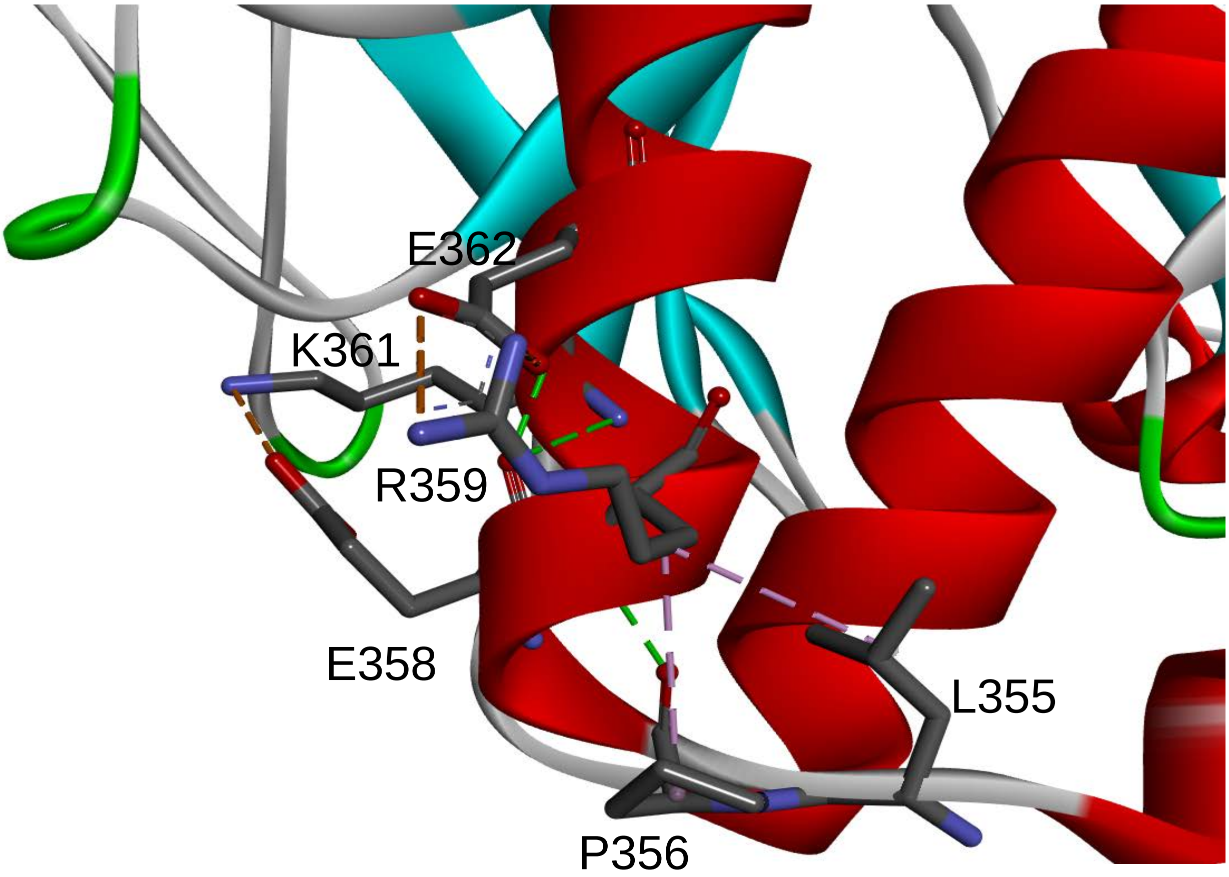

Figure S18. The interaction of R359 and of E362 of MAPK3. MAPK3 active form (pdb: 2ZOQ) is in secondary structure colors. The residues involved in the interactions are depicted in sticks.

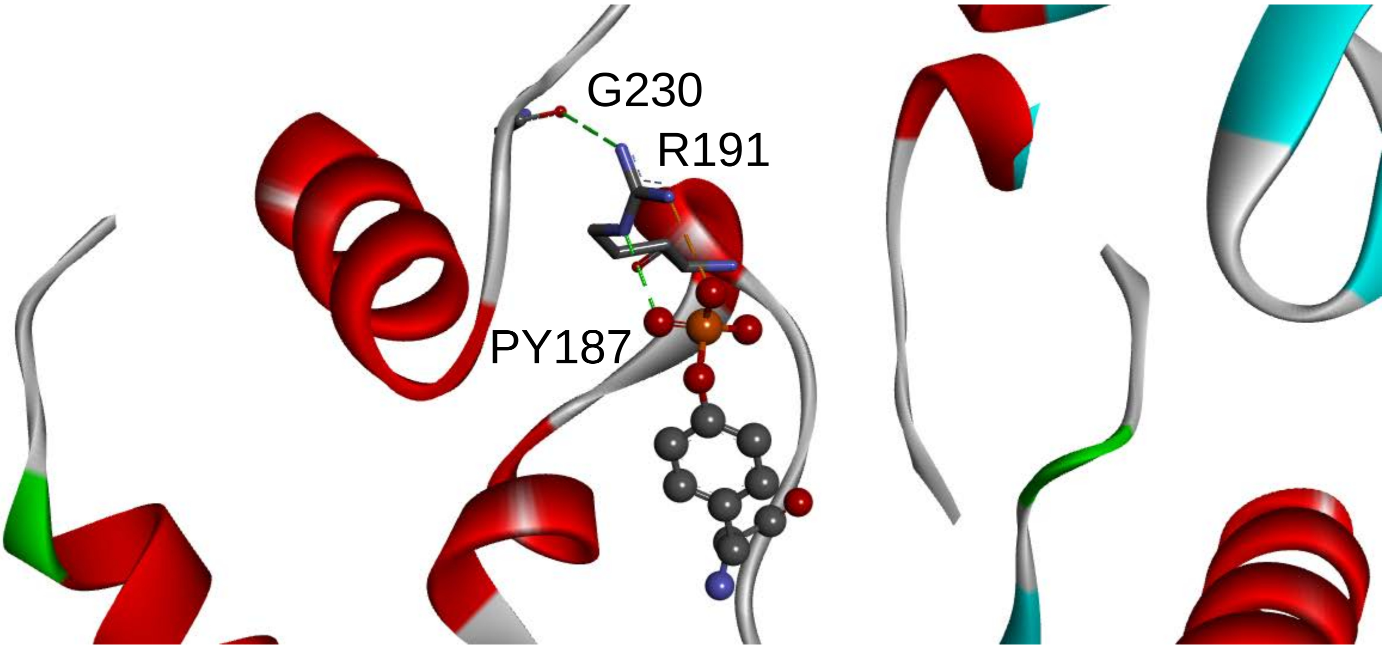

Figure S19. The interaction of R191 in the active form of MAPK1. MAPK1 active form (pdb: TV60) is in secondary structure colors. The residues involved in the interactions are depicted in sticks, phosphotyrosine187 (PY187) is in scaled ball and stick.

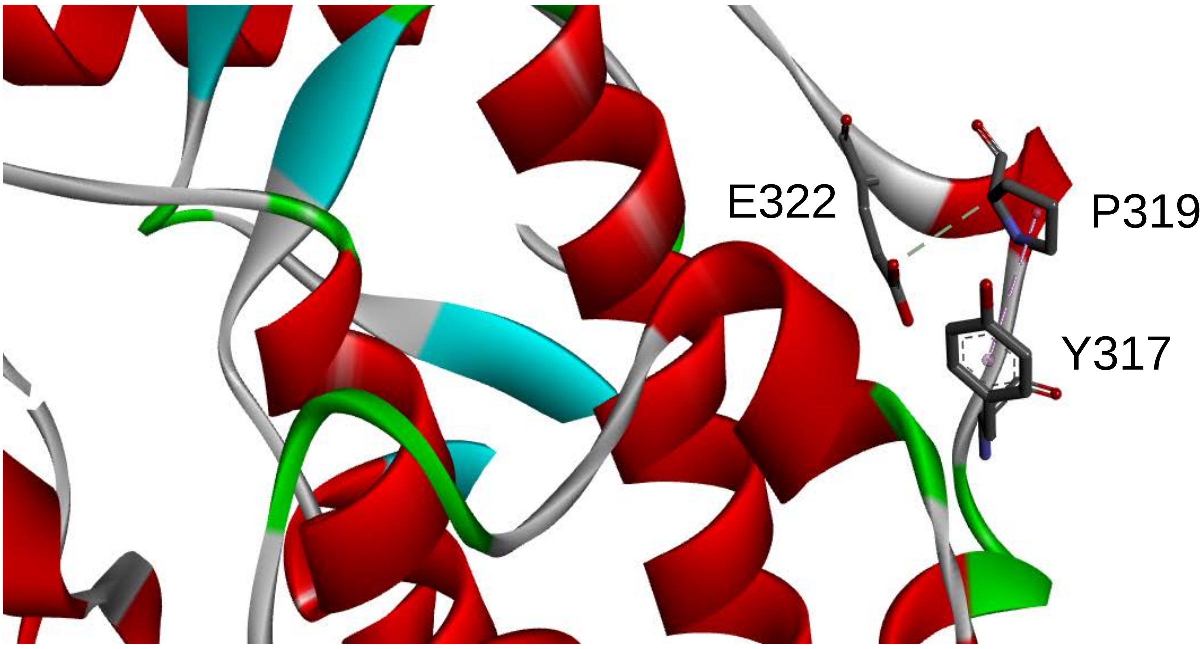

Figure S20. The reciprocal interaction of P319 and E322 in the active form of MAPK1. MAPK1 active form (pdb: TV60) is in secondary structure colors. The residues involved in the interactions are depicted in sticks.

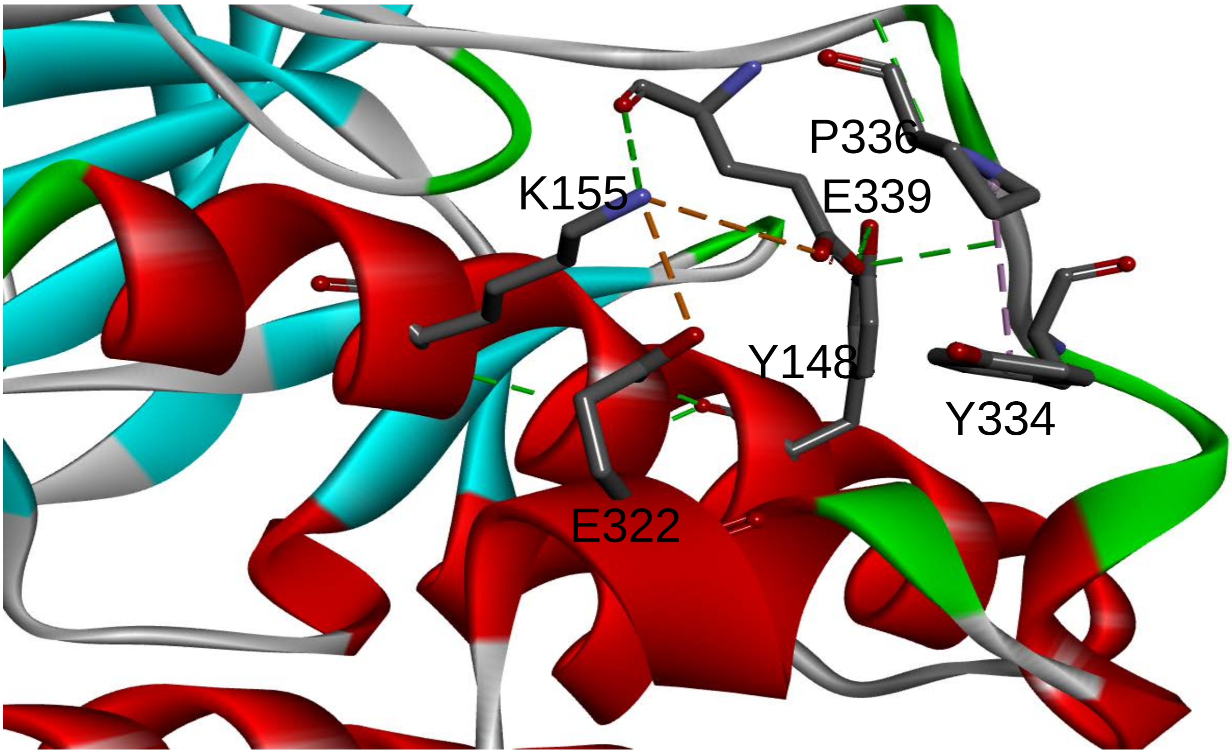

Figure S21. The reciprocal interaction of P336 and E339 in the active form of MAPK3. MAPK3 active form (pdb: 2ZOQ) is in secondary structure colors. The residues involved in the interactions are depicted in sticks.

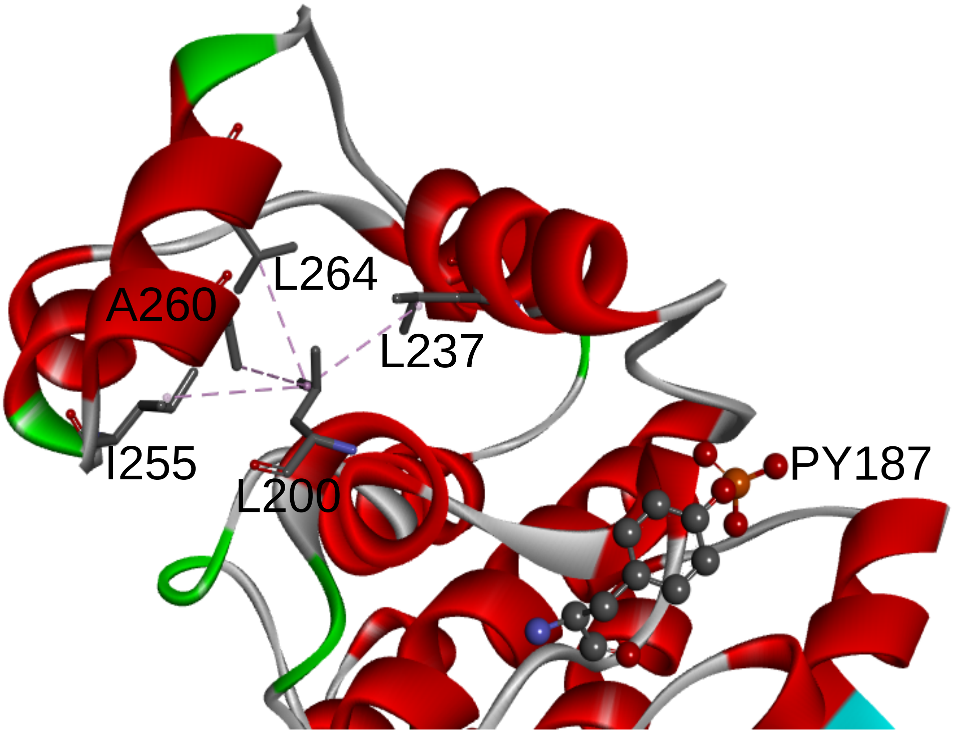

Figure S22. The interaction of L200 in the active form of MAPK1. MAPK1 active form (pdb: 5V60) is in secondary structure colors. The residues involved in the interactions are depicted in sticks, phosphotyrosine187 (PY187) is in scaled ball and stick.
